# The Extracts of *Morinda officinalis* and Its Hairy Roots Attenuate Dextran Sodium Sulfate-Induced Chronic Ulcerative Colitis in Mice by Regulating Inflammation and Lymphocyte Apoptosis

**DOI:** 10.3389/fimmu.2017.00905

**Published:** 2017-08-02

**Authors:** Jian Liang, Jiwang Liang, Hairong Hao, Huan Lin, Peng Wang, Yanfang Wu, Xiaoli Jiang, Chaodi Fu, Qian Li, Ping Ding, Huazhen Liu, Qingping Xiong, Xiaoping Lai, Lian Zhou, Shamyuen Chan, Shaozhen Hou

**Affiliations:** ^1^Guangdong Provincial Key Laboratory of New Chinese Medicinals Development and Research, Guangzhou University of Chinese Medicine, Guangzhou, China; ^2^Shenzhen Fan Mao Pharmaceutical Co., Limited, Shenzhen, China; ^3^Affiliated Huai’an Hospital of Xuzhou Medical University, Huai’an, China; ^4^Section of Immunology, Guangdong Provincial Academy of Chinese Medical Sciences, Guangdong Provincial Hospital of Chinese Medicine, Guangzhou, China

**Keywords:** *Morinda officinalis*, hairy roots culture, ulcerative colitis, anti-inflammatory, immunoregulatory, apoptosis

## Abstract

*Morinda officinalis* is beneficial for the treatment of inflammatory bowel disease (IBD). The hairy root with higher genetic and biochemical stability cultured from *M. officinalis* might have similar effects to treat IBD. In this study, the main chemical composition of the root extracts of *M. officinalis* (MORE) native plant and the hairy root extract of *M. officinalis* (MOHRE) was compared by quantitative HPLC. The difference of their therapeutic effects and potential mechanism was evaluated using 3% dextran sodium sulfate-induced chronic colitis in mice and T lymphocytes *in vitro*. The results found that MOHRE possesses many specific peaks unobserved in the chromatogram of native plant. The content of iridoids in the MORE (3.10%) and MOHRE (3.01%) is somewhat similar but quite different for their anthraquinones’s content (0.14 and 0.66%, respectively). Despite all this, treatment with both MORE and MOHRE significantly attenuated the symptoms of colitis, including diarrhea, body weight loss, colon shortening, histological damage, and decreased inflammatory cytokine levels. In addition, they dose-dependently increased the apoptosis of T lymphocyte *in vivo* and *in vitro*. And, the differences for treatment effects on ulcerative colitis (UC) between them both in this study were mostly insignificant. The results demonstrated that the effects of MORE and MOHRE for the treatment of UC are similar, although there are a few difference on their chemical composition, indicating the hairy root cultured from *M. officinalis* might be able to replace its native plant on treatment of UC. The successful derivation of a sustainable hairy root culture provides a model system to study the synthetic pathways for bioactive metabolites, which will make the use of bioreactors to largely produce traditional medicine become reality.

## Introduction

Ulcerative colitis (UC) is a type of inflammatory bowel disease (IBD), which is characterized by chronic and repeated episodes enteropatia (1). Clinically, patients with UC often have diarrhea, abdominal pain, bloody stools, and recurrent UC, which is prone to cause colon cancer (2). The incidence of UC has been increasing annually, especially in the developed countries. Although the pathogenesis of UC is unclear, evidences have suggested that UC is closely associated with the imbalance of the immune response (3). The defect of T cell apoptosis may lead to an uncontrolled T cell activation and proliferation which is a key mechanism to cause an ultraimmune response and the inductions of proinflammatory cytokines (3). The aggressive behavior of the intestinal immune system can cause immune dysfunction, leading to the infiltration of inflammatory cells and production of a large number of proinflammatory cytokines (4), such as tumor necrosis factor α (TNF-α), interleukin-6 (IL-6), and interleukin-17 (IL-17). These proinflammatory cytokines can cause intestinal damage and further aggravate the condition of UC (5). Therefore, the medicines, which can inhibit T cells proliferation (such as steroids and calcineurin inhibitors) or induce T cells apoptosis (such as TNF-α monoclonal antibody), will exert remarkable therapeutic effects for IBD (6, 7). However, long-term use of these drugs will cause some serious adverse reactions and further aggravate the condition (8). Therefore, it is necessary to search for more effective and safe drugs or therapy to treat UC. Complementary therapies seem to have many advantages, such as lower toxicity, more safety and effectiveness, which have absorbed an increasing number of patients who want to try and use this method to treat IBD (9). Chinese herbals, as a kind of complementary therapies, contain various active components in their extracts that could show therapeutic effects on multiple targets of the body (10).

*Morinda officinalis* is a kind of traditional Chinese herbal medicine and natural nutritious food, as one of the four southern medicines of China. *M. officinalis* has been widely used to treat many diseases such as rheumatism, enuresis, and infertility for more than 2,000 years (11). Many studies have found that *M. officinalis* root mainly contains polysaccharides, anthraquinone, and iridoid (12). These components have extensive pharmacological activities, including anti-inflammation, antioxidant, immune-regulatory, and antitumor effect (13). Relative studies have shown that *M. officinalis* effectively reduced the levels of proinflammatory cytokines (14) (e.g., TNF-α, IL-17, IL-6, and IL-8). Accumulated evidence also suggested that *M. officinalis* could effectively treat colitis in mice induced by dextran sodium sulfate (DSS) (15). Therefore, *M. officinalis* may be working as a potential drug to treat UC. However, *M. officinalis* has a long period of cultivation, which is a time-consuming and seasonal planting, and the eradication of medicinal plants has a negative impact on the natural population of *M. officinalis*. The yield of *M. officinalis* is too low to satisfy commercial purposes. Thus, an alternative method of *M. officinalis* production is required to meet the growing demand of this medicine.

Nowadays, the role of plant tissue culture in the production of high-value secondary metabolites is on the rise. By comparing with different cultivation methods, using hairy root cultures have its benefits such as sustained growth cycle, rapid rate of growth, easy to cultivate in hormone-free medium, and obtain higher genetic and biochemical stability (16). Hairy root cultures become a useful method to produce active compound in limited and secure condition. Hairy roots can be achieved by infecting the wounded higher plants with *Agrobacterium rhizogenes* (17). *A. rhizogenes* contains Ri plasmid, which has two functional areas, namely VIR and T-DNA region. When plants were infected by *A. rhizogenes*, the T-DNA in the inhibited state was activated and integrated into the plant genome, which induced the plant cells to produce hairy roots (18).

In the present study, we used the hairy root cultures method to obtain the hairy roots of *M. officinalis*. We devoted to analyze the differences of the major chemical constituents between roots extract of *M. officinalis* (MORE) native plant and hairy roots extract of *M. officinalis* (MOHRE). Based on this, we further investigated the differences of therapeutic effect between MORE and MOHRE on DSS-induced chronic colitis. In addition, we provided a novel insight into the initial mechanism of MORE and MOHRE in chronic colitis protection.

## Materials and Methods

### Drugs and Reagents

*Morinda officinalis* (no. 20150603) was obtained from Shenzhen Lush Pharmaceutical Co., Ltd. Positive drug mesalazin enteric-coated tablets (no. 150308) was bought from Losan Pharma GmbH (Germany). DSS was purchased from MP Biomedicals (MW; 36,000–50,000; MP Biomedicals, Solon, OH, USA). The enzyme-linked immunosorbent assay (ELISA) kits for TNF-α, IL-6, and IL-17 was bought from Huamei (Cusabio Biotech Co. Ltd., China). All plastic materials were purchased from Falcon Labware (Becton-Dickinson, Franklin Lakes, NJ, USA). RPMI Medium 1640, fetal bovine serum (FBS), and phosphate buffer saline (PBS) were obtained from GIBCO Laboratories (Grand Island, NY, USA). Penicillin G/streptomycin, MTT, and dimethyl sulfoxide (DMSO) were purchased from Sigma (St. Louis, MO, USA). Apoptosis Assay Kit was purchased from Lianke Biology Inc. (Hangzhou, China). APC antimouse CD3 antibody, PE/Cy7 antimouse CD4 antibody, and APC antimouse CD8 antibody were purchased from BioLegend (USA). All other chemicals used were analytical grade and were supplied by the Beijing Chemical Agents Company (China).

### Induction of Hairy Roots of *M. Officinalis*

The stem of *M. officinalis* was used as explants for the induction of hairy roots. The excised root segments were preincubated on half-strength MS (MS/2) solid medium for 3 days before infection. *A. rhizogenes* strain MSU440 was cultivated overnight and collected the bacterial suspension of *A. rhizogenes* by centrifugated at 6,000 rpm for 5 min. The explants were soaked in the overnight grown bacterial suspension of *A. rhizogenes* strain MSU440 (OD_600_ = 0.6–0.8) for 15 min and dry blotted on sterile filter paper. The explants were cultured on 1/2 MS-based solid medium in the dark at 28 ± 1°C. After 3 days of cocultivation, the explants were transferred to 1/2 MS-based solid medium of hormone free which containing sodium cefotaxim (300 mg/L) to eliminate the residual bacteria and incubated in the dark at 25 ± 1°C. The healthy hairy roots were obtained and used after once subculture every 2 weeks in this study.

### Preparation of the Plant Extracts

The powder of root *M. officinalis* or hairy roots of *M. officinalis* was heated to reflux for 2 h with 8 times volume 85% alcohol. The extraction was repeated twice. The extracting solution was filtered to remove the residue. And then it was concentrated by rotary evaporator. The concentrate was freeze dried under vacuum. Dry MORE and MOHRE were used to treat mice with colitis.

### HPLC Analyses

The HPLC analyses of MORE and MOHRE were performed by an Agilent 1260 High Performance Liquid Chromatograph System.

The iridoids analysis was done by using a Phenomenex Luna C18 column (250.0 × 4.6 mm, 5.0 µm). The column temperature was 25.00°C. The mobile phases were 0.4% H_3_PO_4_ solution (A) and methanol (B) with the flow rate at 1.000 mL/min. The gradient elution was 0–13 min, A 95.0%; 13–14 min, A 95.0–91.6%; 14–65 min, A 91.6–91.3%; 65–70 min, A 91.3–86.0%; 70–175 min, A 86.0–84.0%; 75–100 min, A 84.0–80.5%; 100–105 min, A 80.5–95.0%; and postrun 5.00 min. The UV detection wave length was 233 nm, and the injection volume was 10.00 µL. The quantitative analysis of iridoids was carried out by the external standard method.

The anthraquinones analysis was done with an Agilent Eclipse XDB-C18 column (150.0 mm × 4.6 mm, 5.0 µm). The column temperature was 30.00°C. The mobile phase was 0.2% H_3_PO_4_ solution (A) and acetonitrile (B) with the flow rate at 1.000 mL/min. The gradient elution was 0–15 min, A 90.0–80.0%; 15–50 min, A 80.0–70.0%; 50–70 min, A 70.0–60.0%; 70–100 min, A 60.0–50.0%; 100–120 min, A 50.0–20.0%; 120–125 min, A 20.0%; 125–130 min, A 20.0–90.0%; and postrun 5.00 min. The UV detection wave length was 277 nm, and the injection volume was 15.00 µL. The quantitative analysis of anthraquinones was carried out by the external standard method.

### Experimental Animals

Adult male and female Kunming (KM) mice (18–22 g) were purchased from the Laboratory Animal Services Center, Guangzhou University of Chinese Medicine (Guangzhou, China). All animals were raised in accordance with the National Institutes of Health Guide for Laboratory animals’ use. The study was approved by the Animal Ethics Committee of Guangzhou University of Chinese Medicine. Animals were housed under standard environment condition of temperature at 20–25°C under a 12 h dark/light cycle and allowed free access to sterilized water and standard food.

### Acute Toxicity Testing

Acute oral toxicity in mice was performed by using the limit dose test of up-and-down procedure according to OECD guideline (OECD 425) (19). Healthy KM mice were randomly assigned to three groups, each group with six mice (three males and three females). A single dose of 5,000 mg/kg of extracts (MORE or MOHRE) were administered orally to each group. Each mouse was continuously observed for 4 h after the treatment. The mice were further observed once daily for 14 days and the number of deaths, body weight and toxic parameters were recorded.

### Inducement of Experimental Colitis

**Figure 1:**
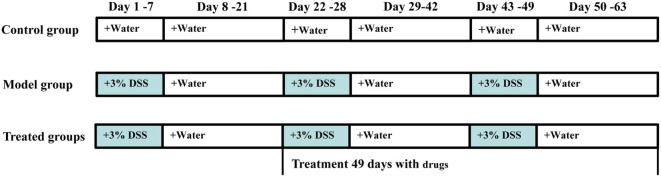
The experimental design for 3% dextran sodium sulfate (DSS)-induced chronic ulcerative colitis (UC) in mice and administration time. Chronic colitis was induced by oral 3% DSS for three cycles. Each cycle was made up of 3% DSS for 7 days followed by 14 days of recovery with water. After the first cycle, modeled mice were randomly divided into eight groups (*n* = 12/group), administration of root extract of *M. officinalis* (MORE) and hairy root extract of *M. officinalis* (MOHRE) to chronic UC in mice for two cycles.

A total of 108 male mice were included in this experiment. Chronic colitis was induced by oral administration of 3% DSS for three cycles (Figure 1), as described by Farkas et al. (20) and Okayasu et al. (21), with slight modification. Randomly selected 12 mice as the normal group, only received tap water every day. The others were induced by three cycles that a 7-day treatment with DSS dissolved in drinking water (3%, w/v) followed by 14 days of recovery with water. After the first cycle, according to their body weight, 96 modeled mice were randomly divided into eight groups (*n* = 12/group): DSS group, mesalazin (50 mg/kg/day) in positive control group, three groups received treatment with MORE (20, 40, and 80 mg/kg/day), the other received treatment with MOHRE (20, 40, and 80 mg/kg/day). The dose selection of extracts was based on clinical practice (usually 6,000 mg/day, 70 kg body weight). Except for the control group, the others were still administered with 3% DSS as described previously, while all drug groups were given the corresponding does of drugs. The treatment lasted for two other cycles and body weight, behavior, stool consistency, fecal occult blood (both visible and occult bleeding), disease activity index (DAI), mortality, food, and water intake of all animals were monitored daily. At the end of the experiment, animals were fasted for 12 h and then anesthetized with pentobarbital sodium (70 mg/kg, i.p.). Blood samples were collected directly from the orbit, which were transferred to 2 mL EP tubes followed by centrifuged at 3,000 rpm for 10 min at 4°C and finally stored at −80°C for biochemical assays. The spleens were weighted and the colons were quickly removed and gently rinsed with sterile and cold saline solution, then moved to cold plate for measuring the length of the colon, colon macroscopic evaluation. Subsequently the colons were divided into two parts, one was fixed in 4% paraformaldehyde for histological examination and the other was stored at −80°C for biochemical assays. Spleen was also fixed in 4% paraformaldehyde for histological examination.

### Assessment of Colitis

#### Disease Activity Index

Disease activity index is determined according to the loss of body weight feces status and macroscopically visible blood in feces, in accordance with the method described by Cooper et al. (22) and Xiao et al. (23).

#### General Morphology Score

Macroscopic damage score of the colon was recorded according to the standard method of Millar et al. (24).

### Histological Examination

Segments of colon and spleen were immediately fixed in 4% paraformaldehyde for 24 h, then washed with water, dehydrated with alcohol, and finally embedded in paraffin. Tissues were cut into standard sections and stained with hematoxylin–eosin (H&E) for histological examination. Segments of colon were evaluated according to the method previously reported by Boirivant et al. (25) and described as follows: 0, normal and no inflammatory cell infiltration; 1, slight inflammatory cell infiltration and no injure in submucosal tissues; 2, moderate inflammatory cell infiltration and submucosal tissues are destroyed (damage range between 10 and 25%); 3, obvious inflammatory cell infiltration, submucosal tissues are destroyed and colonic wall thickening (damage range between 25 and 50%); and 4, serious inflammatory cell infiltration, large scale colon tissue damage (damage range > 50%) and colonic wall thickening.

### Measurement of Cytokines

The systemic levels of TNF-α, IL-6, and IL-17 in the serum were measured by using a commercially available ELISA according to the manufacturer’s instructions.

### Apoptosis Evaluation by Annexin-V/PI *In Vivo*

At the end of the experiment, the splenocytes and peripheral blood (PB) lymphocytes were isolated from colitis mice. The method was described previously by Su et al. (26). The cells (2 × 10^6^ cells/mL) were stained for surface markers, antimouse CD3-APC, antimouse CD4-PE/Cy7, or antimouse CD8-APC in PBS for 20 min at RT in the dark. After washing, cells apoptosis were detected by the AnnexinV/PI detection kit, according to manufacturer’s instruction, briefly, cells were washed twice with precold PBS, then suspended in binding buffer and stained with 5 µL annexin V-FITC and 10 µL PI for 15 min at 25°C in the dark. Annexin V^+^/PI^−^ cells were considered early apoptotic cells. Annexin V^+^/PI^+^ cells were considered late apoptotic cells. Apoptosis analysis was detected by FACS canto™ flow cytometer.

### Cell Preparation

Splenocytes were isolated as described previously by Su et al. (26), with slight modification. Mice were sacrificed and the spleens were aseptically separated, washed with precold PBS. The spleens were ground and through a 70 µm strainer. After centrifuge at 300 × *g* for 5 min at 4°C, ACK Lysis Buffer was used to remove erythrocytes. Cells were collected by centrifuged at 300 × *g* for 5 min at 4°C and washed twice with PBS. Splenocytes were resuspended and cultured in RPMI1640 medium supplemented with 10% (v/v) FBS, penicillin (100 U/mL), and streptomycin (100 µg/mL) under a humidified 5% (v/v) CO_2_ atmosphere at 37°C. Cells viability analysis was assessed by using trypan blue dye exclusion staining and all cases cell viability was higher than 95%.

### Cytotoxicity Assay

Cytotoxicity assay was evaluated by MTT assay, 100 µL of splenocytes were seeded in 96-well plates at 3 × 10^6^ cells/mL, treated with 100 µL MORE and MOHRE (50–400 µg/mL) for 24 h, respectively. After that, 20 µL of MTT (5 mg/mL) was added to each well and further incubated for 4 h at 37°C with 5% CO_2_. 150 µL of DMSO was added into 96-well plates, and finally the plates were read at 490 nm with Multiskan Go. Without cytotoxic and optimal doses of MORE and MOHRE were selected for further apoptosis assessments. The formula was applied to calculate the cell viability (%):

$$\text{Cell viability}(\%)=\frac{\text{(}A_{S}-A_{b})}{\text{(}A_{c}-A_{b})}\times 100$$

where *A_s_, A_c_*, and *A_b_* represent the absorbance of the different concentrations wells of drugs, control wells, and blank wells, respectively.

### Measurement of Cell Proliferation

Splenocytes were separated and cultured by the same methods in the section cell preparation. 100 µL of splenocytes were seeded in 96-well plates at 3 × 10^6^ cells/mL. The cells were stimulated with 5 µg/mL of Concanavalin A (ConA) and treated with 100 µL MORE and MOHRE (50, 100, and 200 µg/mL) for 24 h, the range of concentrations was optimal values according to the cytotoxicity. The formula was applied to calculate the cell proliferation by the same methods in the section Cytotoxicity Assay.

### Apoptosis Analysis *In Vitro*

Splenocytes were seeded in 48-well plates at 3 × 10^6^ cells/mL, treated with different concentrations of MORE and MOHRE (50, 100, and 200 µg/mL) for 4 h following by 5 µg/mL ConA stimulation or no stimulation with ConA for another 24 h. Apoptosis analysis was tested according to the method in section apoptosis evaluation by Annexin-V/PI *in vivo*. Apoptosis analysis was detected by FACS canto™ flow cytometer and fluorescence staining.

### Statistical Analysis

Statistical analysis was performed using SPSS software (version 18.0; SPSS, Chicago, IL, USA). The analysis of survival was conducted using Kaplan–Meier method (log-rank test). All results are expressed as mean ± SEM. Results are using one-way ANOVA or Student’s *t*-test when appropriate. Values of *p* < 0.05 were considered statistically significant.

## Results

### HPLC Analysis of *M. officinalis* and Hairy Roots of *M. officinalis*

**Figure 2:**
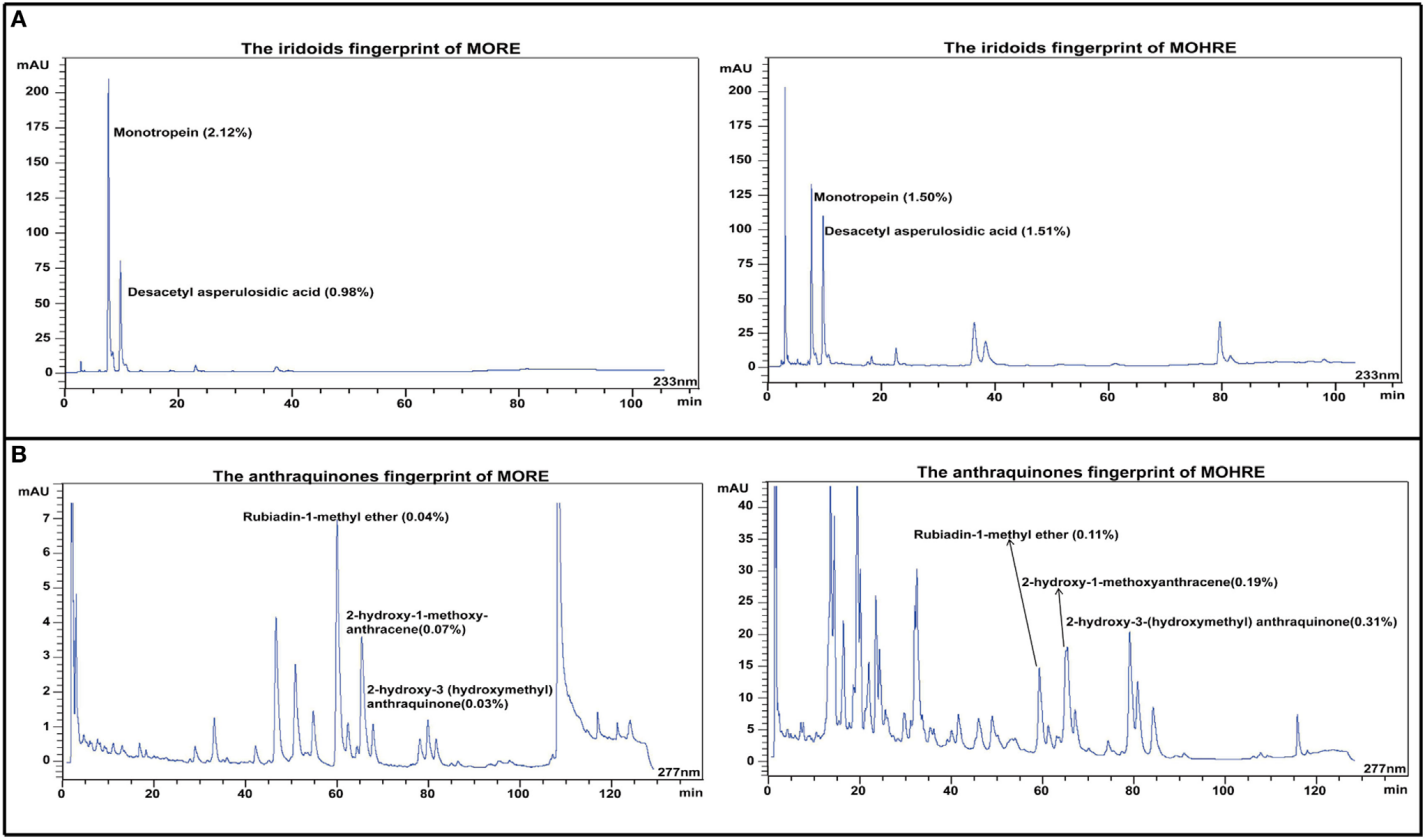
The HPLC fingerprints derived from the roots extract of *Morinda officinalis* (MORE) and hairy roots extract of *Morinda officinalis* (MOHRE). **(A)** The HPLC fingerprints of iridoids; **(B)** The HPLC fingerprints of anthraquinones.

Iridoid and anthraquinones are the main active ingredients of *M. officinalis*. Here, the chromatographic peaks of hairy roots extract of *M. officinalis* (MOHRE) were more abundant than roots extract of *M. officinalis* (MORE) (Figure 2), suggesting that there were more secondary metabolites in the MOHRE extracts, especially anthraquinones. The content of monotropein is 2.12 and 1.50% in MORE and MOHRE, respectively, while the content of Desacetyl asperulosidic acid is 0.98% in MORE and 1.51% in MOHRE. In addition, the contents of rubiadin-1-methyl ether, 2-hydroxy-1-methoxy-anthracene and 2-hydroxy-3 (hydroxymethyl) anthraquinone are 0.04, 0.07, and 0.03% in MORE, as well as the contents of rubiadin-1-methyl ether, 2-hydroxy-1-methoxy-anthracene, and 2-hydroxy-3(hydroxymethyl)anthraquinone are 0.11, 0.19, and 0.31% in MOHRE. Suggesting that the content of active iridoid is similar to MORE and MOHRE, the anthraquinones are derived from the MOHRE is higher than MORE.

### The Acute Toxicity Study of MORE and MOHRE

Roots extract of *Morinda officinalis* and MOHRE did not caused obvious toxic reactions and mortality with the dose of 5,000 mg/kg by oral administration. In addition, during the 14 days observation time, all animals looked bright and the body weight did not have significant change (Table 1).

**Table 1:** Effects of MORE and MOHRE in mice after acute oral administration.

Dose (g/kg)	Animals		Effects		
	Dead/total treated mice	Mortality (%)	Symptoms of toxicity	Body weight change (%)	
Control	0/6	0	None	Female	125.9 ± 0.44
				Male	130.7 ± 2.46
5 (MORE)	0/6	0	None	Female	125.1 ± 1.52
				Male	129.3 ± 3.44
5 (MOHRE)	0/6	0	None	Female	126.3 ± 0.91
				Male	130.4 ± 2.51

*Values represent mean ± SEM of three animals*.

### MORE and MOHRE Attenuated DSS-Induced Murine Experimental Chronic Colitis

**Figure 3:**
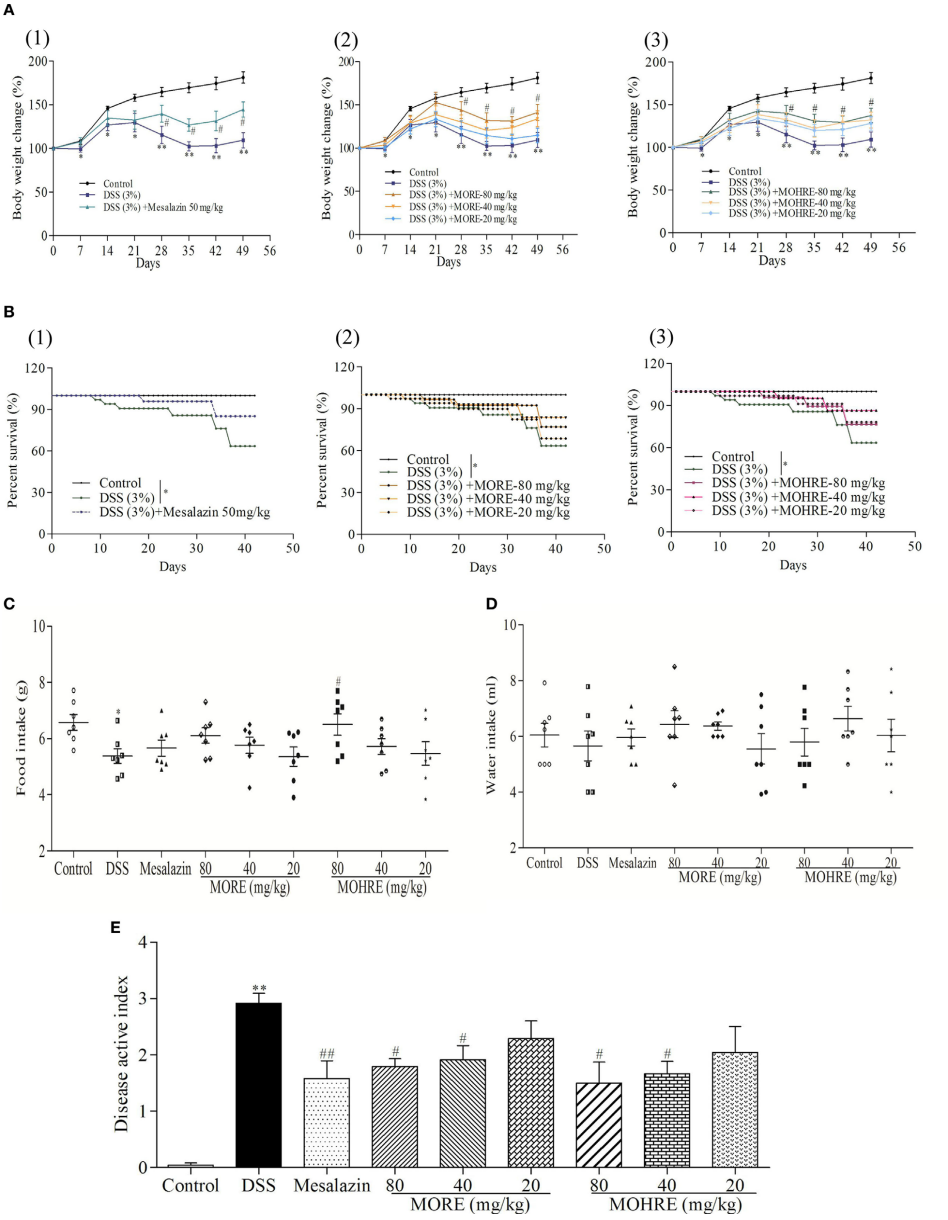
The therapeutic effect of root extract of *Morinda officinalis* (MORE) and hairy root extract of *M. officinalis* (MOHRE) ameliorated dextran sodium sulfate (DSS)-induced chronic ulcerative colitis (UC) in mice. **(A)** Body weight. **(B)** Survival rate. **(C)** Food intake. **(D)** Water intake. **(E)** Disease active index. All data are presented as mean ± SEM of six to nine mice. *p < 0.05. ^**^p < 0.01 vs. control group; *^#^p* < 0.05. *^##^p* < 0.01 vs. DSS group.

In this study, the body weight of colitis mice induced by DSS decreased significantly from day 7, and the animal condition continued to deteriorate until the end of the experiment in DSS group. All drug-administration groups significantly restored body weight (Figure 3A). The enteritis induced by DSS significantly increased the mortality of animals. Treatment with mesalazin as a positive drug, MORE, and MOHRE reduced the death of animals (Figure 3B). In addition, food intake and water intake also were reduced in DSS group. Treatment with mesalazin, MORE, and MOHRE could improve food intake and water intake to a certain extent (Figures 3C,D). DAI is a most important indicator of the effect evaluation in IBD model. In this study, the DAI score increased significantly in DSS group, indicating a severe condition of colitis. By oral administration of mesalazin, MORE, and MOHRE, the DAI scores were decreased significantly (Figure 3E). The roles of MORE and MOHRE revealed a dose-dependent manner. And there were not obvious differences on reversing these pathological symptoms of UC between MORE and MOHRE under the same concentration. These results showed that both MORE and MOHRE could attenuate DSS-induced experimental chronic UC with similar effect.

**Figure 4:**
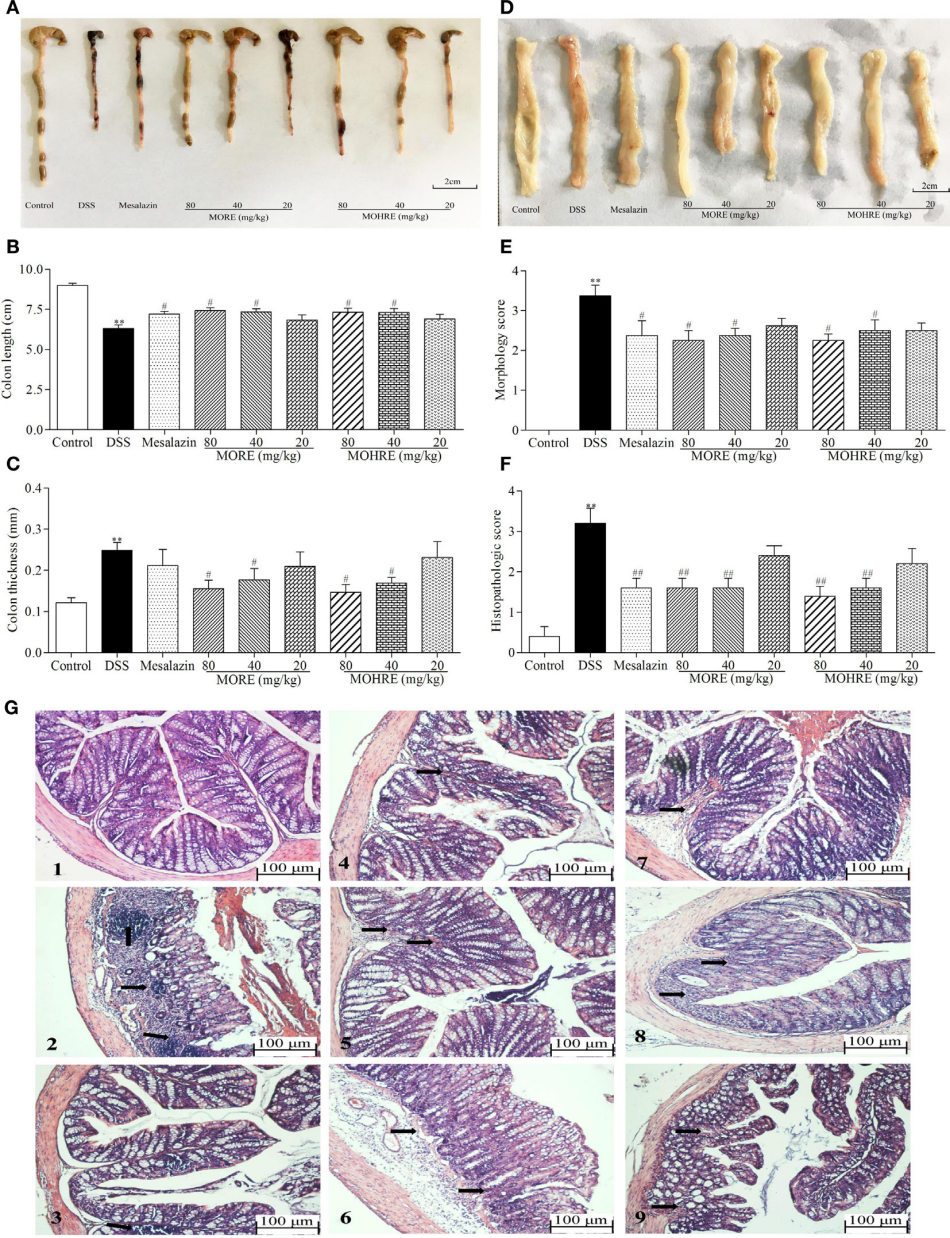
Root extract of *Morinda officinalis* (MORE) and hairy root extract of *M. officinalis* (MOHRE) ameliorated colonic injury in dextran sodium sulfate (DSS)-induced chronic colitis mice. **(A)** Representative colon from each group. **(B)** Colon length. **(C)** Colon thickness. **(D)** The representative pictures of colon macroscopic appearances in each group. **(E)** Morphology score. **(F)** Histological score. **(G)** Representative hematoxylin–eosin (H&E) staining of colon tissues of each group (black arrows indicating inflammatory infiltration and injury), (1) Control group, (2) DSS group, (3) DSS + mesalazin (positive drug group), (4) DSS + MORE 80 mg/kg, (5) DSS + MORE 40 mg/kg, (6) DSS + MORE 20 mg/kg, (7) DSS + MOHRE 80 mg/kg, (8) DSS + MOHRE 40 mg/kg, and (9) DSS + MOHRE 20 mg/kg. All data are presented as mean ± SEM of six to nine mice. *p < 0.05. ^**^p < 0.01 vs. control group; *^#^p* < 0.05. *^##^p* < 0.01 vs. DSS group.

The colon length is inversely associated with the severity of DSS-induced colitis. Our results showed that the colon from mice in mesalazin, MORE, and MOHRE groups is longer than in the DSS group (Figures 4A,B). Colon thickness and morphology score are also the important indicators to evaluate the therapeutic effect of drug. We found that mesalazin, MORE, and MOHRE reduced colon thickness and morphology score (Figures 4C–E). In addition, the H&E staining of colonic tissues revealed the anti-inflammatory effects of mesalazin, MORE, and MOHRE in colon. Compared with the normal structure of the colon, DSS group showed obvious inflammatory cell infiltration, absence of epithelial cells and goblet cells. However, significant reductions in the inflammatory cell infiltration and mucous membrane ulcer were observed after treatment with mesalazin, MORE, and MOHRE (Figures 4F,G). Furthermore, these positive effects of MORE and MOHRE displayed a dose-dependent manner. An insignificant difference between MORE and MOHRE was observed at the same concentration. These results indicated that both MORE and MOHRE could effectively protect DSS-induced colon injury in mice.

### The Effect of MORE and MOHRE on the Level of Inflammatory Cytokines in DSS-Induced Colitis

**Figure 5:**
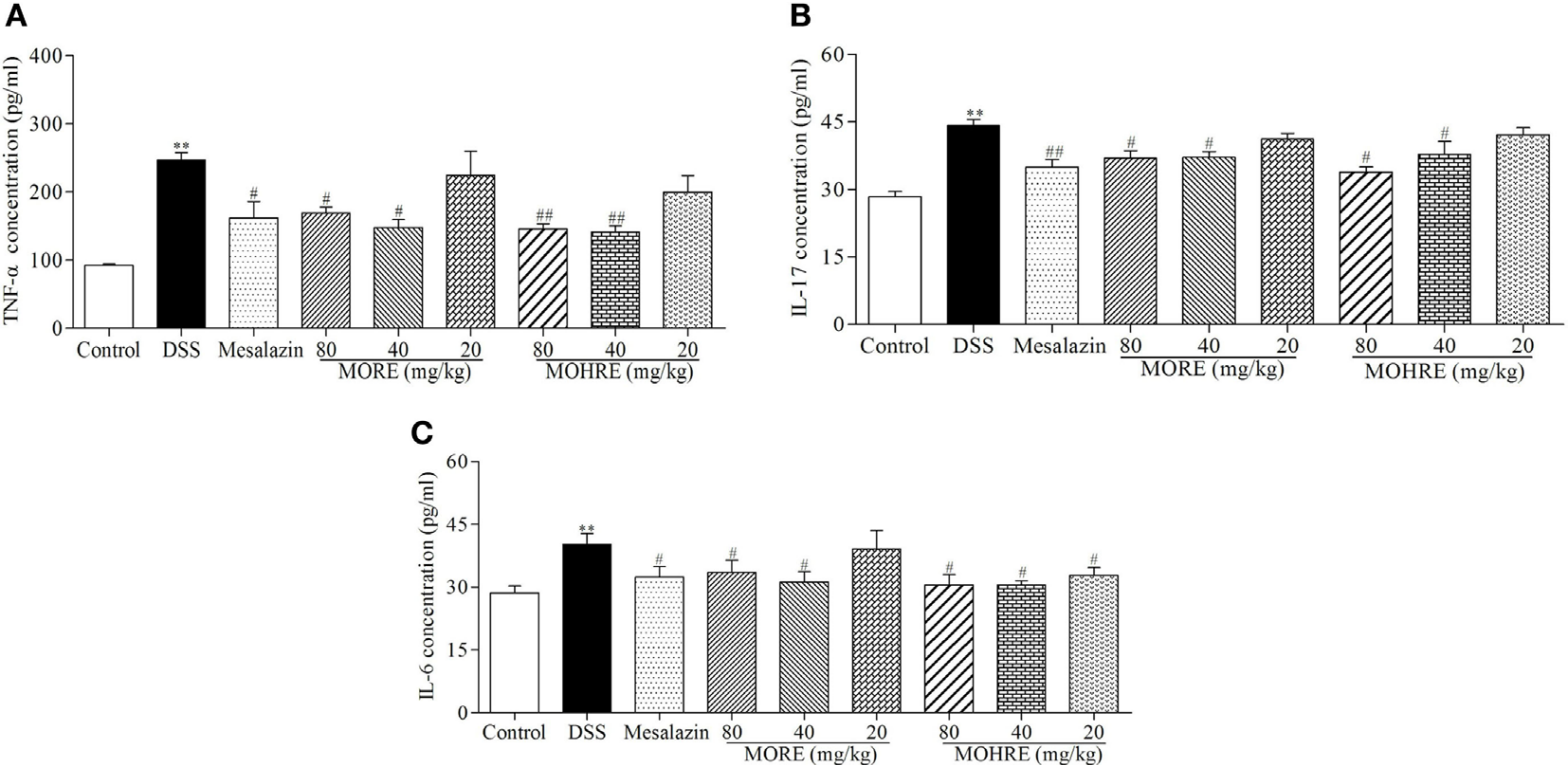
The effect of root extract of *Morinda officinalis* (MORE) and hairy root extract of *M. officinalis* (MOHRE) on proinflammatory cytokines production in the serum of mice with dextran sodium sulfate (DSS)-induced chronic colitis. **(A)** Tumor necrosis factor α (TNF-α) concentration. **(B)** Interleukin (IL)-17 concentration. **(C)** IL-6 concentration. All data are expressed as mean ± SEM of six mice. *p < 0.05. ^**^p < 0.01 vs. control group; *^#^p* < 0.05. *^##^p* < 0.01 vs. DSS group.

It is well known that inflammation plays an important role in the pathogenesis of UC. The abnormal activation of immune system can cause excessive secretions of proinflammatory cytokines. To understand the anti-inflammatory effect of MORE and MOHRE in DSS-induced chronic UC in mice, we measured the serum levels of proinflammatory cytokines TNF-α, IL-6, and IL-17 were significantly increased in DSS group, which were all decreased significantly in mesalazin, MORE, and MOHRE groups (Figure 5). There are still no significant differences between MORE and MOHRE.

### The Protective Effect of MORE and MOHRE on Spleen in DSS-Induced Chronic Colitis

**Figure 6:**
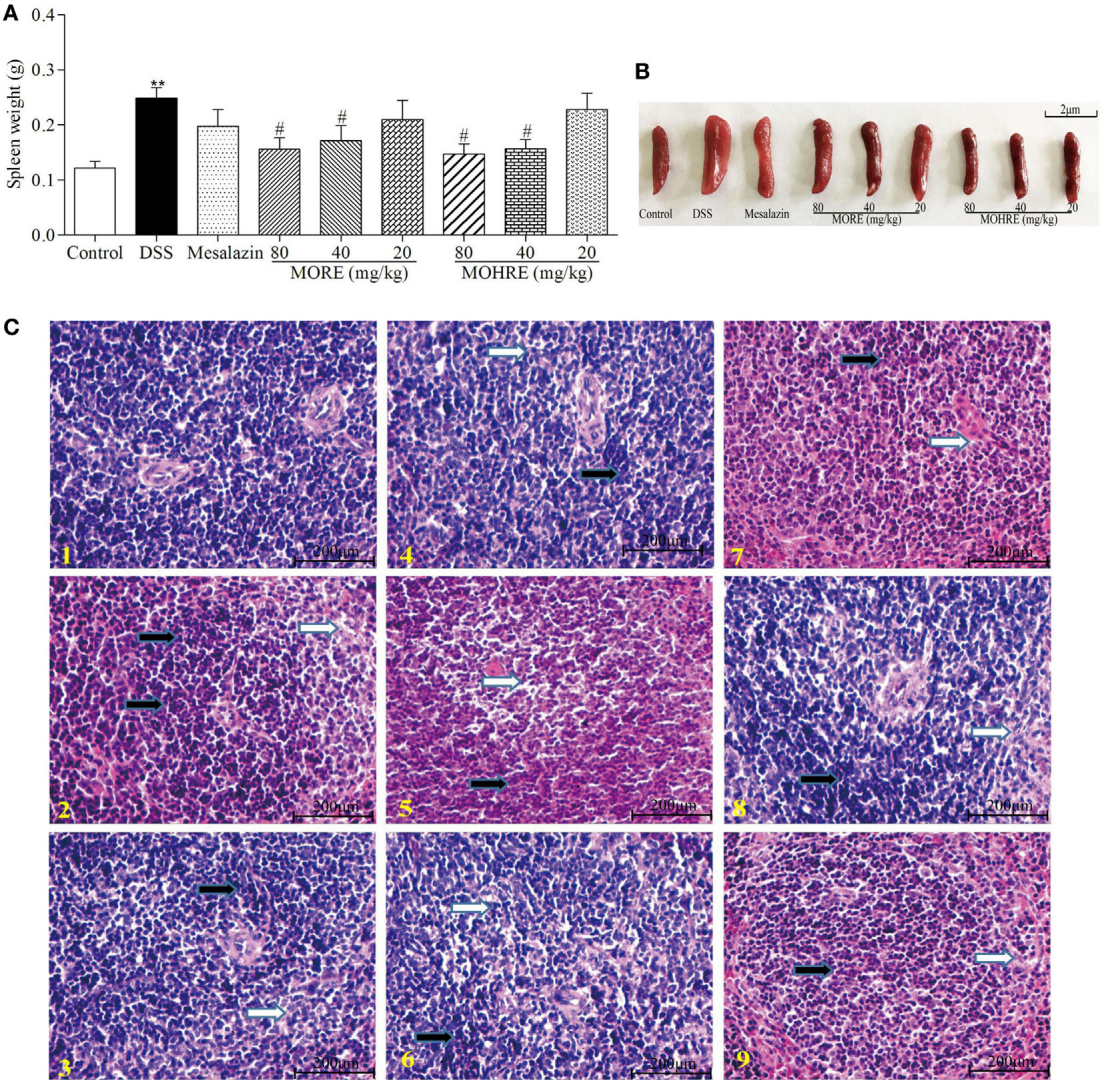
The protective effect of root extract of *Morinda officinalis* (MORE) and hairy root extract of *M. officinalis* (MOHRE) on spleen in DSS-induced chronic colitis mice. **(A)** Spleen weight. **(B)** Representative spleen weight from each group. **(C)** Representative hematoxylin–eosin (H&E) staining of spleen tissues of each group (black arrows indicating inflammatory cell accumulation and white arrows indicating injury). (1) Control group, (2) DSS group, (3) DSS + mesalazin (positive drug group), (4) DSS + MORE 80 mg/kg, (5) DSS + MORE 40 mg/kg, (6) DSS + MORE 20 mg/kg, (7) DSS + MOHRE 80 mg/kg, (8) DSS + MOHRE 40 mg/kg, (9) DSS + MOHRE 20 mg/kg. All data are expressed as mean ± SEM of seven mice. *p < 0.05. **p < 0.01 vs. control group; ^#^*p* < 0.05. ^##^*p* < 0.01 vs. DSS group.

Chronic colitis often causes the disorder of immune function. In this study, we found that oral administration of DSS significantly increases spleen weight, and MORE and MOHRE significantly inhibited spleen swelling (Figures 6A,B). In addition, we analyzed the degree of spleen damage by H&E staining. We also found that DSS led to the change of spleen structure, swelling, and the accumulation of inflammatory cells. However, MORE and MOHRE could significantly effectively alleviate the injury of the spleen (Figure 6C). This protective effect between MORE and MOHRE was not significantly different.

### MORE and MOHRE Induced the Apoptosis of Splenocytes, but Not PB Lymphocytes in Colitis Mice

**Figure 7:**
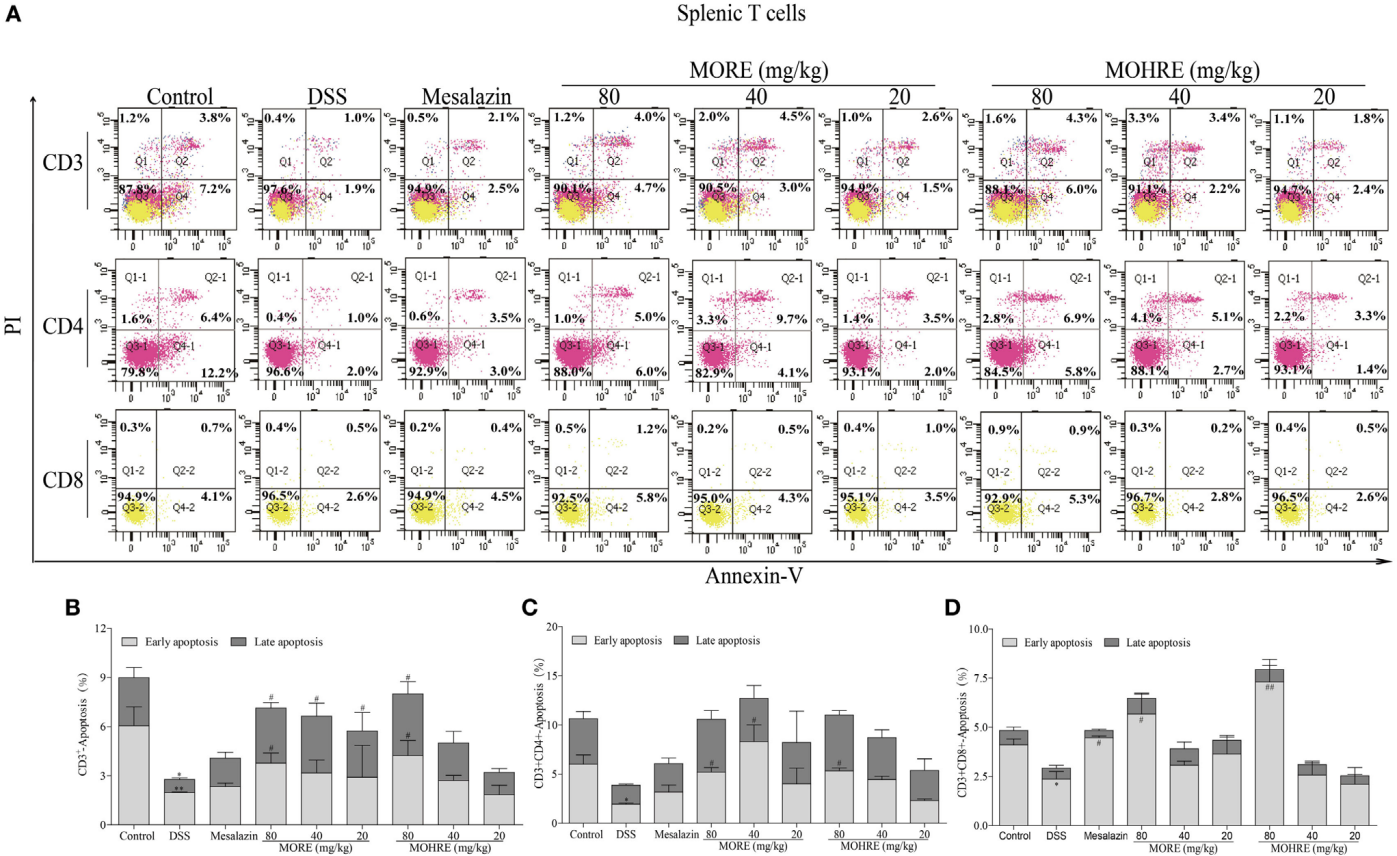
The apoptosis effect of root extract of *Morinda officinalis* (MORE) and hairy root extract of *M. officinalis* (MOHRE) on splenic T lymphocytes in dextran sodium sulfate (DSS)-induced chronic colitis mice. **(A)** The apoptosis of CD3^+^, CD4^+^, and CD8^+^ T cells was detected by Annexin V/PI staining. **(B)** The apoptosis ratio of CD3^+^ T cells. **(C)** The apoptosis ratio of CD3^+^CD4^+^ T cells. **(D)** The apoptosis ratio of CD3^+^CD8^+^ T cells. All data are expressed as mean ± SEM of three mice. *p < 0.05. ^**^p < 0.01 vs. control group; *^#^p* < 0.05. *^##^p* < 0.01 vs. DSS group.

**Figure 8:**
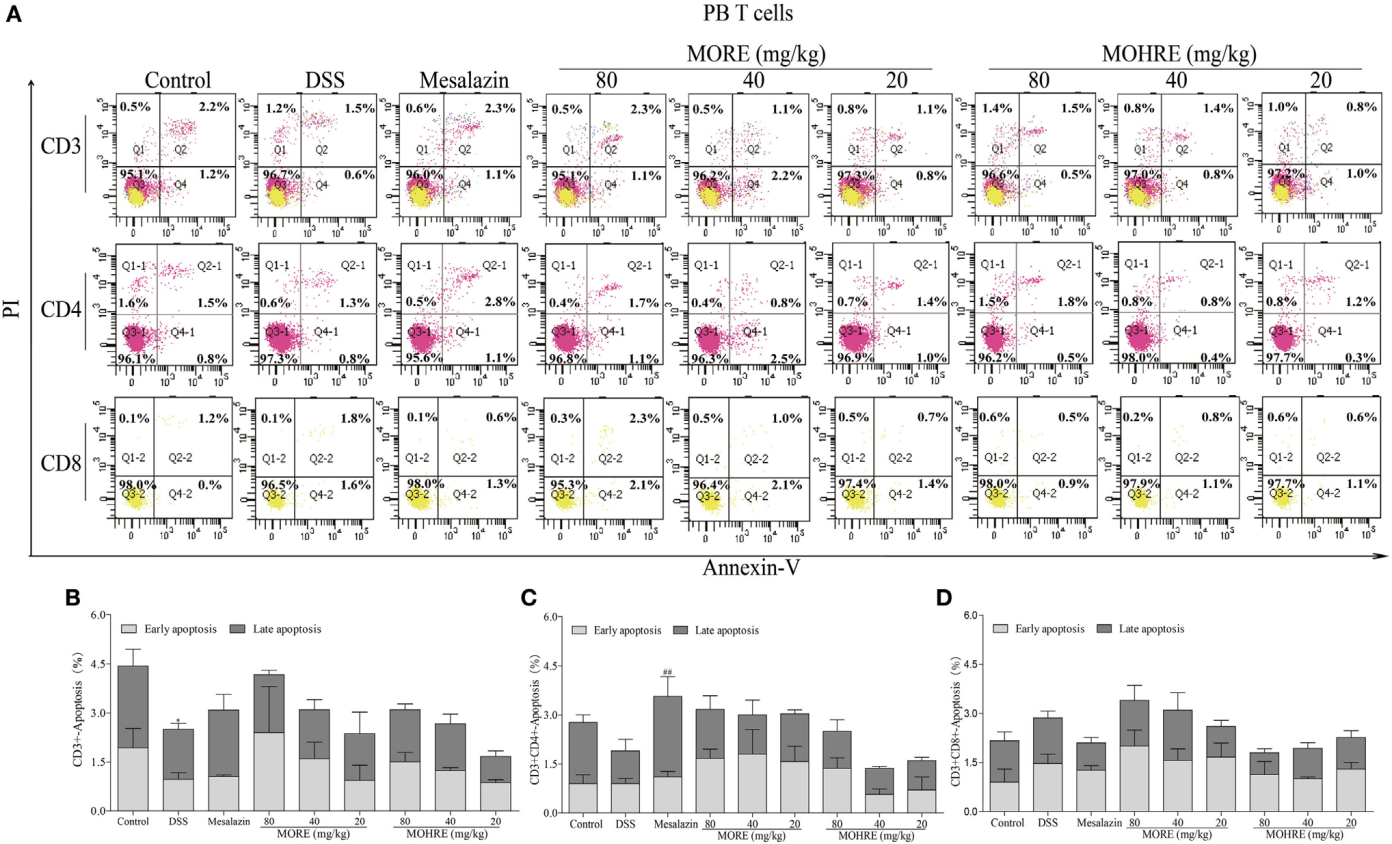
The apoptosis effect of root extract of *Morinda officinalis* (MORE) and hairy root extract of *M. officinalis* (MOHRE) on peripheral blood (PB) T lymphocytes in dextran sodium sulfate (DSS)-induced chronic colitis mice. **(A)** The apoptosis of CD3^+^, CD4^+^ and CD8^+^ T cells was detected by Annexin V/PI staining. **(B)** The apoptosis ratio of CD3^+^ T cells. **(C)** The apoptosis ratio of CD3^+^CD4^+^ T cells. **(D)** The apoptosis ratio of CD3^+^CD8^+^ T cells. All data are expressed as mean ± SEM of three mice. *p < 0.05. ^**^p < 0.01 vs. control group; *^#^p* < 0.05. *^##^p* < 0.01 vs. DSS group.

The apoptosis of T cells has been confirmed to be critically involved in the pathogenesis of UC. In the present study, splenocytes and PB lymphocytes from chronic colitis mice were analyzed by flow cytometry after two cycle’s treatment of MORE and MOHRE. To analyze the subsets of splenic and PB T cells that suffer apoptosis following MORE and MOHRE treatment in chronic colitis mice, we applied annexin V^+^/PI^+^ among total T cell (CD3^+^), helper T cells (CD3^+^CD4^+^) and cytotoxic T cell (CD3^+^CD8^+^) in spleen and PB. We found that the early and late apoptosis of splenic CD3^+^ T were significantly decreased in DSS treated mice when compared with the control group, and early apoptosis of splenic CD4^+^ and CD8^+^ T cells were also significantly decreased in DSS group. Meanwhile, except PB CD3^+^ T cells were significantly reduced in late apoptosis, the apoptosis of PB CD4^+^ and CD8^+^ T cells had no difference in DSS group when compared with the control group. Interestingly, MORE and MOHRE significantly induced the early and late apoptosis of splenic CD3^+^ T cells in a dose-dependent manner *in vivo*. In addition, the early apoptosis of splenic CD4^+^ and CD8^+^ T cells in colitis mice were also significantly increased. However, there is no significantly difference in the induction of PB T cells apoptosis after treatment with MORE and MOHRE (Figures 7 and 8). The data showed that MORE and MOHRE could obviously induce the apoptosis of splenocytes, but not PB lymphocytes in chronic colitis mice. Meanwhile, the apoptosis effect of MOHRE on splenocytes or PB lymphocytes *in vivo* is similar to MORE.

### The Cytotoxic and Proliferous Effect of MORE and MOHRE to Splenic Lymphocytes

**Figure 9:**
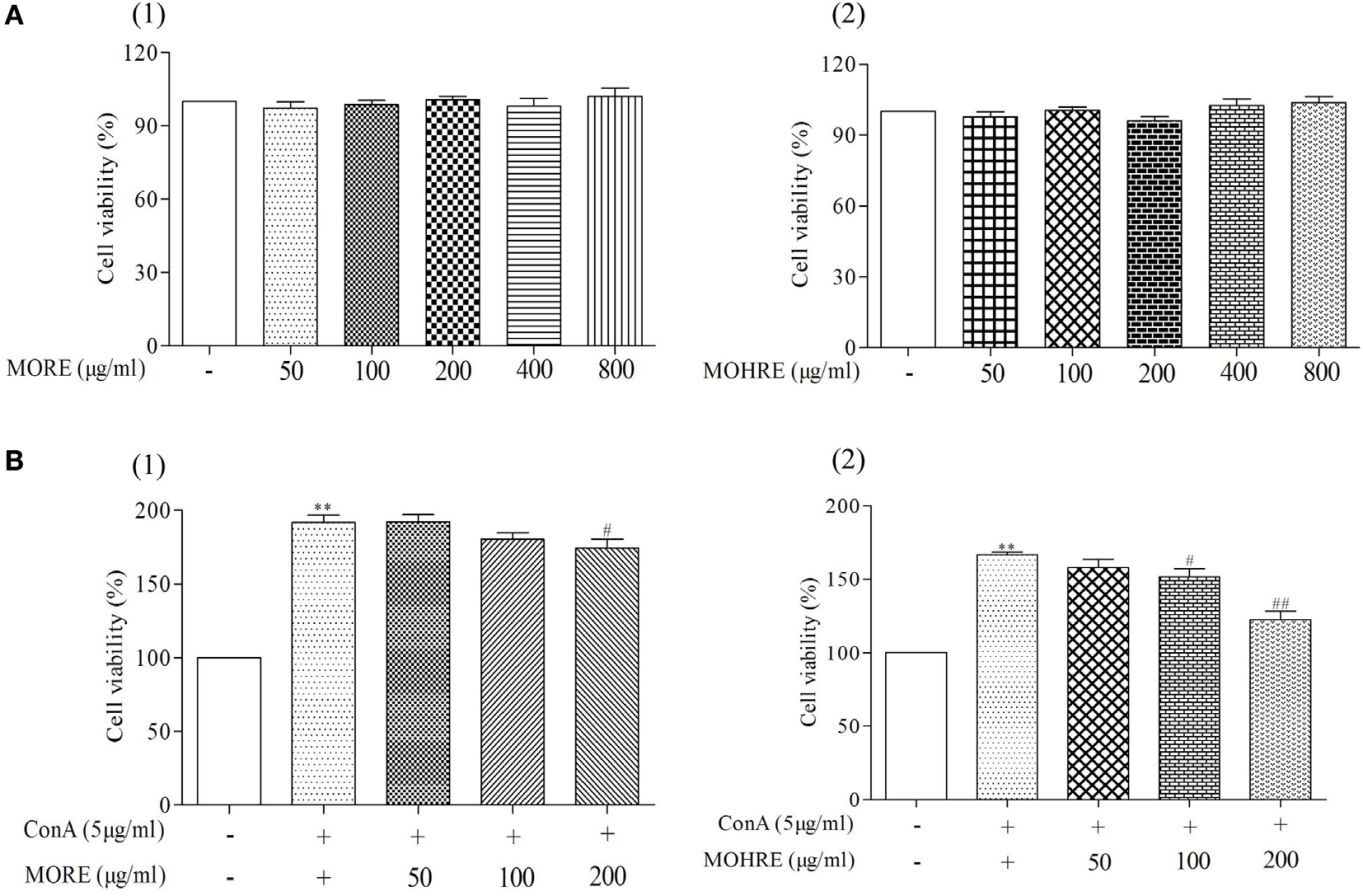
Cytotoxic and proliferous effect of root extract of *Morinda officinalis* (MORE) and hairy root extract of *M. officinalis* (MOHRE) on lymphocytes. Cell viability was measured using MTT assay with different concentration (50–800 µg/mL) of MORE and MOHRE in the presence or absence of Concanavalin A (ConA) for 24 h. **(A)** Cytotoxicity of MORE and MOHRE on lymphocytes from normal mice in the absence of ConA for 24 h. **(B)** The proliferous effect of MORE and MOHRE on lymphocytes in the presence of ConA for 24 h. All data are expressed as mean ± SEM of three independent experiments. *p < 0.05. ^**^p < 0.01 vs. control group; *^#^p* < 0.05. *^##^p* < 0.01 vs. ConA group.

According to the results from *in vivo* study, we found that splenic lymphocytes were much more sensitive to MORE- and MOHRE-induced apoptosis. Here, we further investigated the effect of MORE and MOHRE on splenic lymphocytes *in vitro*. The different concentrations of MORE and MOHRE were added into splenic lymphocytes in different 96-well plates for 24 h. The data showed that MORE and MOHRE had no toxicity to splenic lymphocytes (Figure 9A). However, after ConA stimulation, MORE and MOHRE inhibited the proliferation of splenic lymphocytes in a dose-dependent manner (Figure 9B), possessing unsignificant difference between them at the same concentration. The results revealed that MORE and MOHRE exert immunoregulatory properties for the proliferation of ConA-activated T cells *in vitro*.

### The Apoptosis Assay of MORE and MOHRE on Splenic T Cells *In Vitro*

**Figure 10:**
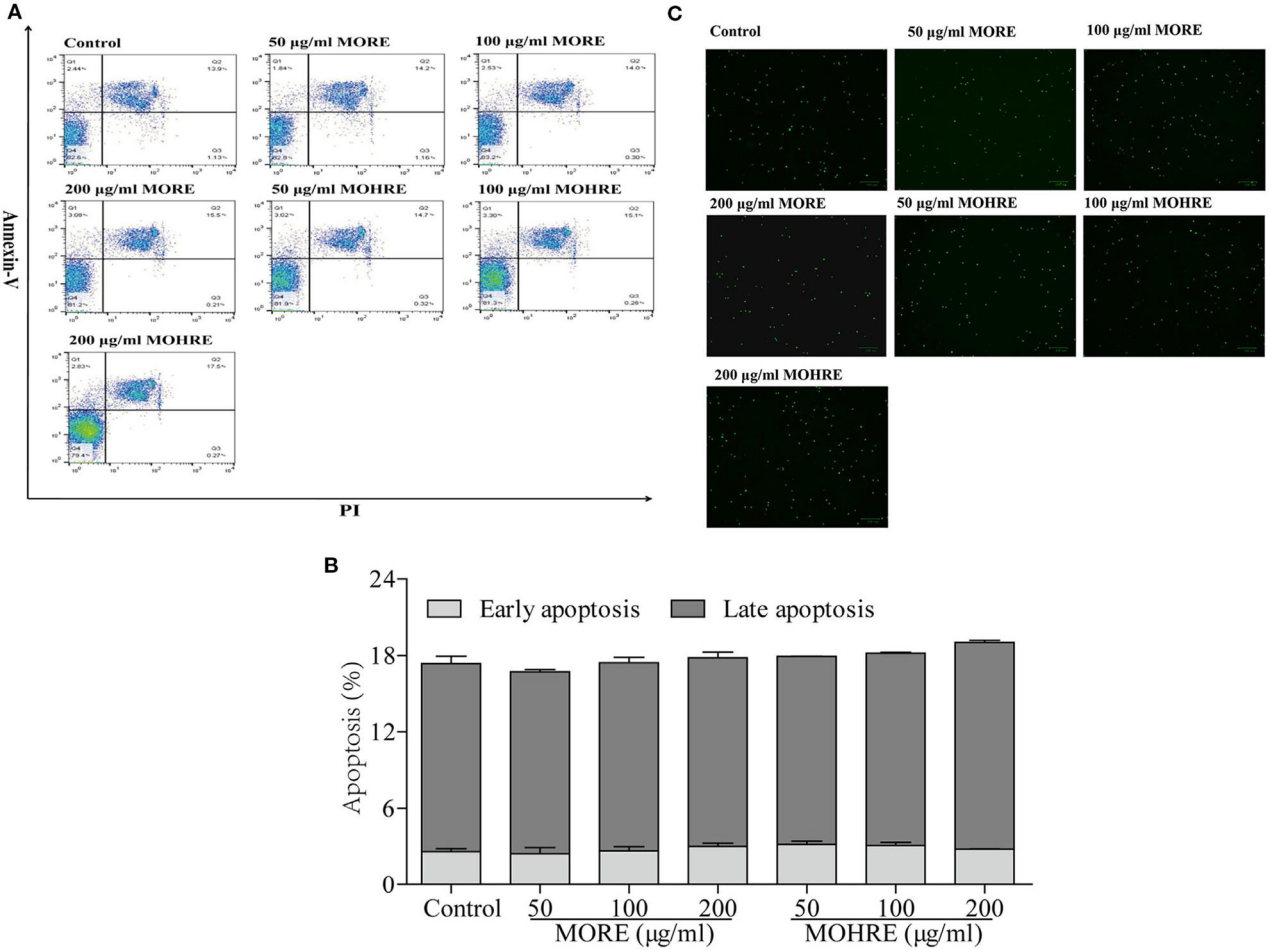
The apoptosis effect of root extract of *Morinda officinalis* (MORE) and hairy root extract of *M. officinalis* (MOHRE) on normal splenic lymphocytes. **(A)** Representative FACS picture in each group. **(B)** Apoptosis assay. **(C)** Representative images were obtained using a fluorescence microscope (100 µm). All data are expressed as mean ± SEM of three independent experiments. *p < 0.05. ^**^p < 0.01 vs. control group.

**Figure 11:**
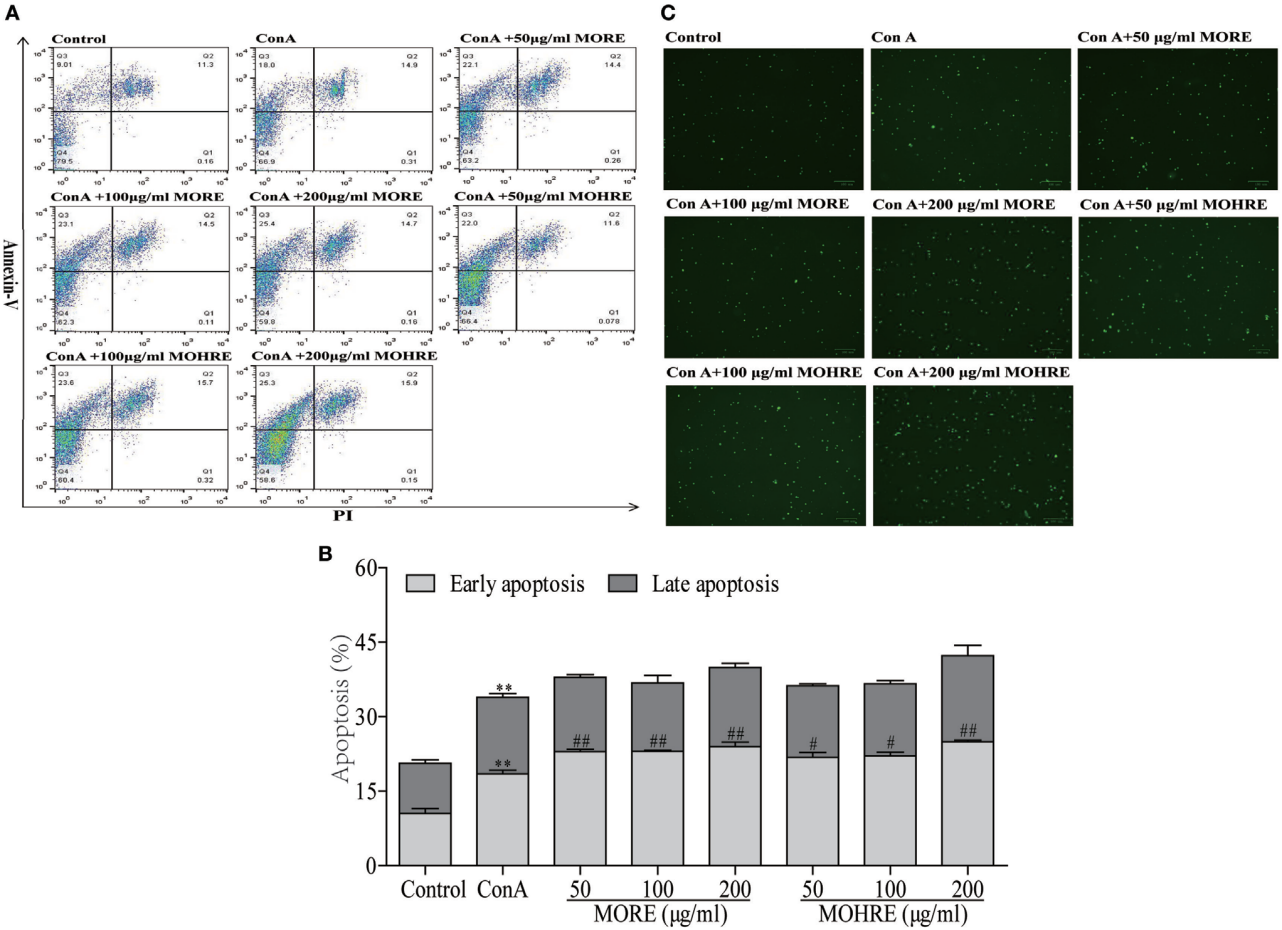
The apoptosis effect of root extract of *Morinda officinalis* (MORE) and hairy root extract of *M. officinalis* (MOHRE) on spleen lymphocytes have been activated by Concanavalin A (ConA). **(A)** Representative FACS picture in each group. **(B)** Apoptosis assay. **(C)** Representative images were obtained using a fluorescence microscope (100 µm). All data are expressed as mean ± SEM of three independent experiments. *p < 0.05. **p < 0.01 vs. control group; *^#^p* < 0.05. *^##^p* < 0.01 vs. ConA group.

**Figure 12:**
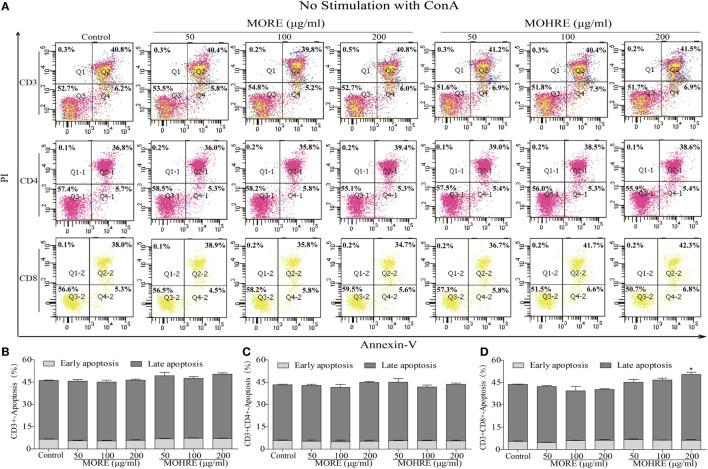
The apoptosis effect of root extract of *Morinda officinalis* (MORE) and hairy root extract of *M. officinalis* (MOHRE) on CD3^+^, CD4^+^, and CD8^+^ T cells in the absence of Concanavalin A (ConA) stimulation. **(A)** The apoptosis of CD3^+^, CD4^+^, and CD8^+^ T cells was detected by Annexin V/PI staining. **(B)** The apoptosis ratio of CD3^+^ T cells. **(C)** The apoptosis ratio of CD3^+^CD4^+^ T cells. **(D)** The apoptosis ratio of CD3^+^CD8^+^ T cells. All data are expressed as mean ± SEM of three independent experiments. *p < 0.05. ^**^p < 0.01 vs. control group.

**Figure 13:**
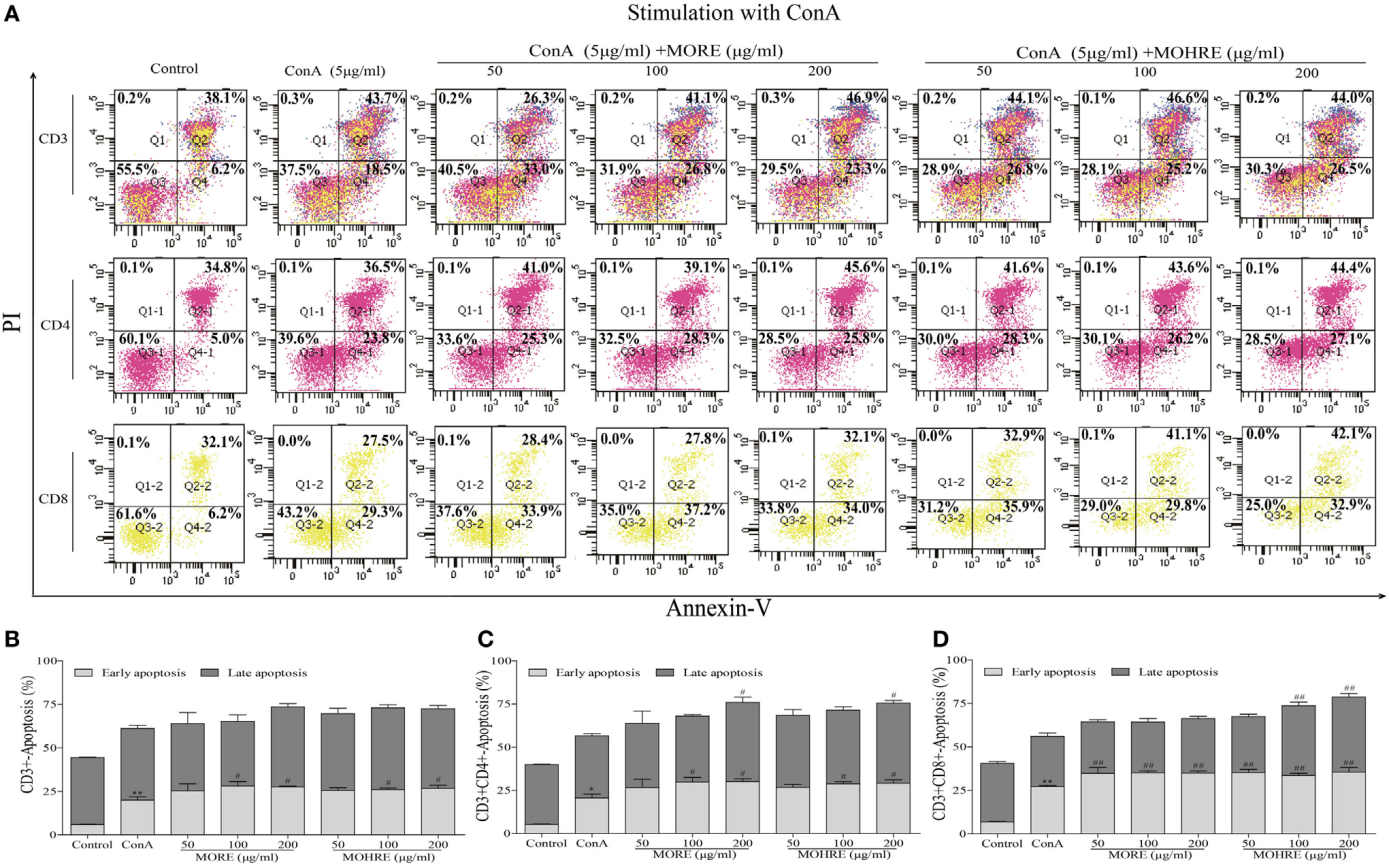
The apoptosis effect of root extract of *Morinda officinalis* (MORE) and hairy root extract of *M. officinalis* (MOHRE) on CD3^+^, CD4^+^, and CD8^+^ T cells in the presence of Concanavalin A (ConA) stimulation. **(A)** The apoptosis of CD3^+^, CD4^+^, and CD8^+^ T cells was detected by Annexin V/PI staining. **(B)** The apoptosis ratio of CD3^+^ T cells. **(C)** The apoptosis ratio of CD3^+^CD4^+^ T cells. **(D)** The apoptosis ratio of CD3^+^CD8^+^ T cells. All data are expressed as mean ± SEM of three independent experiments. *p < 0.05. ^**^p < 0.01 vs. control group; *^#^p* < 0.05. *^##^p* < 0.01 vs. ConA group.

To explore the relationship between the inhibition of splenic T cells proliferation and apoptosis, we incubated splenic lymphocytes with MORE and MOHRE in the presence or absence of ConA. Without ConA stimulation, MORE and MOHRE were unable to significantly induce lymphocytes apoptosis (Figure 10). However, after stimulation with ConA for 24 h, both MORE and MOHRE can dose-dependently induce the apoptosis of ConA-activated T lymphocytes (Figure 11). Meanwhile, MORE and MOHRE also could induced the early apoptosis of activated T lymphocytes at 48 in the presence of ConA stimulation (Presentation S1 in Supplementary Material). In addition, we further to explore whether CD3^+^, CD4^+^, and CD8^+^ T cells population is susceptible to cause apoptosis by MORE and MOHRE in both unstimulated and Con A-stimulated lymphocyte. Our data indicated that the early and late apoptosis ratios of CD3^+^, CD4^+^, and CD8^+^ T cells were no statistically differences in the unstimulated lymphocyte (Figure 12). However, the lymphocyte activated with ConA and exposed to MORE and MOHRE could dose-dependently increased the early and late apoptosis ratios of CD3^+^, CD4^+^, and CD8^+^ T cells (Figure 13). The difference of these effects between MORE and MOHRE was not significant at the same concentration.

## Discussion

In the present study, we investigated the differences of the major chemical constituents between MORE and MOHRE. Based on this, we demonstrated the therapeutic effect of the hairy roots of *M. officinalis* on DSS-induced chronic UC in mice and investigated the apoptosis effect of *M. officinalis* and its hairy roots on T lymphocytes *in vivo* and *in vitro* for the first time. Our results showed that *M. officinalis* and its hairy roots could protect against DSS-induced chronic colitis and inhibit abnormal activation of T cells via inducing apoptosis *in vivo* and *in vitro*.

*Morinda officinalis* native plant contains much more polysaccharides than *M. officinalis* hairy roots (data not shown). Thus we used 85% ethanol to generate extracts without polysaccharides, the present data showed that both MORE and MOHRE are effective in treating UC mice, indicating that polysaccharides in MORE may not be an essential component in the treatment of UC mice. The content of iridoids in MORE is similar to that in MOHRE, both contain up to 3%. While the content of anthraquinones in MORE is obvious less than that in MOHRE, the ratio is 0.14 vs 0.66%. Meanwhile, we found that the MOHRE exert a similar therapeutic effect on DSS-induced chronic UC in mice. Several studies have shown that these iridoids in *M. officinalis* have anti-inflammatory and immunomodulatory properties (27, 28). It also has been reported that monotropein, the main iridoid in *M. officinalis*, has therapeutic effect on DSS-induced acute colitis mice with a dose up to 200 mg/kg (15), which is much higher than the dose of MORE and MOHRE we used in our study. These data further indicated that besides monotropein, other iridoids and anthraquinone in *M. officinalis* could also exert an important role in the treatment of UC.

People with UC often have symptoms such as diarrhea, bloody stool, abdominal pain, body weight loss, and colonic shortening (29). These clinical parameters are essential to assess the severity of UC and evaluate the efficacy of the potential drugs. In this study, the results showed that oral administration of MORE and MOHRE could significantly improve body weight loss, food intake, fecal status, intestinal bleeding, and colonic tissue injury on 3% DSS-induced chronic UC in mice. In addition, DSS induced colonic mucosa injury, which led to colon edema and atrophy (30). Colonic shortening and DAI score are used as important indicators to evaluating colonic injury (31). In the present study, treatment with MORE and MOHRE significantly inhibited the colonic shortening and reduced DAI score in colitis mice. These data showed that MORE and MOHRE protected against DSS-induced chronic colitis in mice.

The DSS-induced colitis model exerted colonic mucosa injury, inflammatory cell infiltration, goblet cell loss, and colonic wall thickness (32). Many studies have demonstrated that neutrophil infiltration in the colonic mucosa tissues is one of the most remarkable histological features observed in patients with UC (33, 34). Diffuse neutrophil infiltration in the colonic mucosa tissues could aggravate inflammatory infiltration (35). Therefore, reducing the amount of neutrophil can effectively alleviate the colon injury. According to the histological analysis, oral administration of MORE and MOHRE could significantly alleviate colonic tissue damage, neutrophil infiltration, and promotes the repair of colonic tissue on DSS-induced colitis. The pharmacological effects may be attributed to their extracts containing iridoids and anthraquinones. Related studies have shown that these chemical compositions can downregulate NF-κB signaling pathways, indicating that a significant anti-inflammatory effect (36).

The abnormal expression of proinflammatory cytokines not only occurs in IBD patients but also appears in DSS-induced colitis model. TNF-α is a major proinflammatory cytokines, its abnormal expression is closely related to the immune dysfunction (37, 38). The aggressive release of TNF-α can activate the intestinal adaptive immune system, and recruit a large number of neutrophils and macrophages to infiltrate the colonic mucosa tissues, which in turn leads to colon injury (39). Therefore, many immunosuppressive agents have been developed to decrease the levels of TNF-α for the treatment of UC. In addition, IL-6 and IL-17 also participated in the inflammatory response of IBD and induced the production of many other proinflammatory cytokines. Overexpression of these cytokines plays an important role in the pathogenesis of IBD (40). In the present study, the levels of TNF-α, IL-6, and IL-17 were tested in colitis mice. The data show that oral administration of MORE and MOHRE can significantly decrease the serum levels of TNF-α, IL-6, and IL-17 in colitis mice, suggesting that MORE and MOHRE could suppress the production of proinflammatory cytokines and then relieve the inflammatory response.

T lymphocytes are important for immune system homeostasis and host defense, which can secrete proinflammatory and anti-inflammatory cytokines (41, 42). When the body is injured, immune cells would produce relevant cytokines to maintain immune homeostasis. However, the over expansion of activated lymphocytes can lead to a series of autoimmune diseases (43), including rheumatoid arthritis, atopic illnesses, and IBD. Many studies have indicated that the excessive proliferation of lymphocytes is closely related to the pathogenesis of IBD (44). Inhibiting the proliferation of lymphocytes or inducing lymphocytes apoptosis may provide the basis for a potential therapeutic strategy in patients with UC (45, 46). Accumulated evidences have showed that the induction of CD4^+^ and CD8^+^ T cells apoptosis is beneficial to treat colitis (44, 47). The data *in vivo* study indicated that MORE and MOHRE could significantly inhibit splenomegaly, improve the damage of spleen, and decrease the production of inflammatory cytokines in DSS-induced chronic colitis mice. The effects may be related to its immunomodulatory effects. The proliferation of activated T cells and malformation in cell death regulation play an important role in the pathogenesis of IBD including UC and CD. Meanwhile, many immunosuppressive agents have been used to treat IBD due to the induction of T cell apoptosis (6, 48). Based on this, we further investigated the apoptosis effects of MORE and MOHRE on splenic lymphocytes and PB lymphocytes *in vivo*. Interestingly, MORE and MOHRE significantly increased the early apoptosis of splenic CD3^+^, CD4^+^, and CD8^+^ T cells. The late apoptosis of splenic T cells only appeared in CD3^+^ T cells after treatment with MORE and MOHRE. However, the induction of T cells apoptosis in PB T cells was no significant differences. These results showed that MORE and MOHRE exert effects against activated T cells by the induction of apoptosis and the effects are more sensitive to splenic lymphocytes apoptosis.

To further confirm the apoptosis effects of MORE and MOHRE on T lymphocytes, we tested the apoptosis effects of MORE and MOHRE on T cells in the presence or absence of ConA stimulation. We found that MORE and MOHRE were non-toxic to T cells and did not induce the apoptosis of T cells in the absence of ConA stimulation. However, both MORE and MOHRE significantly inhibited the proliferation of ConA-activated T lymphocytes. Meanwhile, MORE and MOHRE dose-dependently induced activated T cells apoptosis in early and late stage. The early apoptosis of cells is a reversible course when the apoptotic stimulation is cleaned up (49). With the rise of early apoptosis, irreversible late apoptosis would gradually be accumulated. In this study, the results *in vitro* were consistent with *in vivo*. MORE and MOHRE were more sensitive to induce early apoptosis of CD3^+^, CD4^+^, and CD8^+^ T cell in the imbalance of immune status. These results from *in vitro* study further showed that MORE and MOHRE could exert the immunoregulatory effect by induction of T cells apoptosis only when the immune dysfunction. In this sense, it is possible that the therapeutic effects of MORE and MOHRE on DSS-induced chronic colitis may be achieved via inducing apoptosis of overactivated lymphocytes, leading to the decrease of the levels of inflammatory cytokines and the restoration of normal immune response. Based on this, it is also worth to further investigate the apoptosis effect of MORE and MOHRE on Treg or Teff cells *in vivo and in vitro*. To explore this, we should mark additional surface markers of Treg or Teff cells. Unfortunately, it was technically impossible that a 5-color flow cutometer was used in this study. However, further study along these lines should be continued.

## Conclusion

Although the content of active chemical composition is not completely consistent, our study discovered that the hairy roots of *M. officinalis* are equally effective as the native plant in the treatment of UC in a DSS-induced chronic colitis model. Our research demonstrated three possible mechanisms of action for MORE and MOHRE, including the anti-inflammatory effect, the promotion of T lymphocyte apoptosis and the reduction of abnormal immune responses. In addition, the successful derivation of a sustainable hairy root culture provides a model platform to study the synthetic pathways for bioactive metabolites such as iridoids and anthraquinones and makes the use of bioreactors to largely produce traditional Chinese medicine a reality to treat IBD.

## Ethics Statement

Adult male and female Kunming (KM) mice (18–22 g) were purchased from the Laboratory Animal Services Center, Guangzhou University of Chinese Medicine (Guangzhou, China). All animals are raised in accordance with the National Institutes of Health Guide for Laboratory animals’ use. The study was approved by the Animal Ethics Committee of Guangzhou University of Chinese Medicine. Animals were housed under standard environment condition of temperature at 20–25°C under a 12 h dark/light cycle, and allowed free access to sterilized water and standard food.

## Author Contributions

JL, PW, HL, and JL contributed to the animal experiments, cell experiments, and data analysis. HH, YW, CF, XJ, HL, and QX contributed to the study analysis and data analysis. XL and LZ contributed to the revision of the manuscript. SC and SH contributed to the conception and design of the study.

## Conflict of Interest Statement

The authors declare that the research was conducted in the absence of any commercial or financial relationships that could be construed as a potential conflict of interest.
