# Corrigendum: The Extracts of *Morinda officinalis* and Its Hairy Roots Attenuate Dextran Sodium Sulfate-Induced Chronic Ulcerative Colitis in Mice by Regulating Inflammation and Lymphocyte Apoptosis

**DOI:** 10.3389/fimmu.2020.02092

**Published:** 2020-09-11

**Authors:** Jian Liang, Jiwang Liang, Hairong Hao, Huan Lin, Peng Wang, Yanfang Wu, Xiaoli Jiang, Chaodi Fu, Qian Li, Ping Ding, Huazhen Liu, Qingping Xiong, Xiaoping Lai, Lian Zhou, Shamyuen Chan, Shaozhen Hou

**Affiliations:** ^1^Guangdong Provincial Key Laboratory of New Chinese Medicinals Development and Research, Guangzhou University of Chinese Medicine, Guangzhou, China; ^2^Shenzhen Fan Mao Pharmaceutical Co., Limited, Shenzhen, China; ^3^Affiliated Huai'an Hospital of Xuzhou Medical University, Huai'an, China; ^4^Section of Immunology, Guangdong Provincial Academy of Chinese Medical Sciences, Guangdong Provincial Hospital of Chinese Medicine, Guangzhou, China

**Keywords:** *Morinda officinalis*, hairy roots culture, ulcerative colitis, anti-inflammatory, immunoregulatory, apoptosis

In the original article, there was a mistake in Figure 10C as published. One image (MORE, 200 μg/ml) was mistakenly duplicated from another image (Control) during the figure preparation. The corrected Figure 10 appears below.

**Figure 10:**
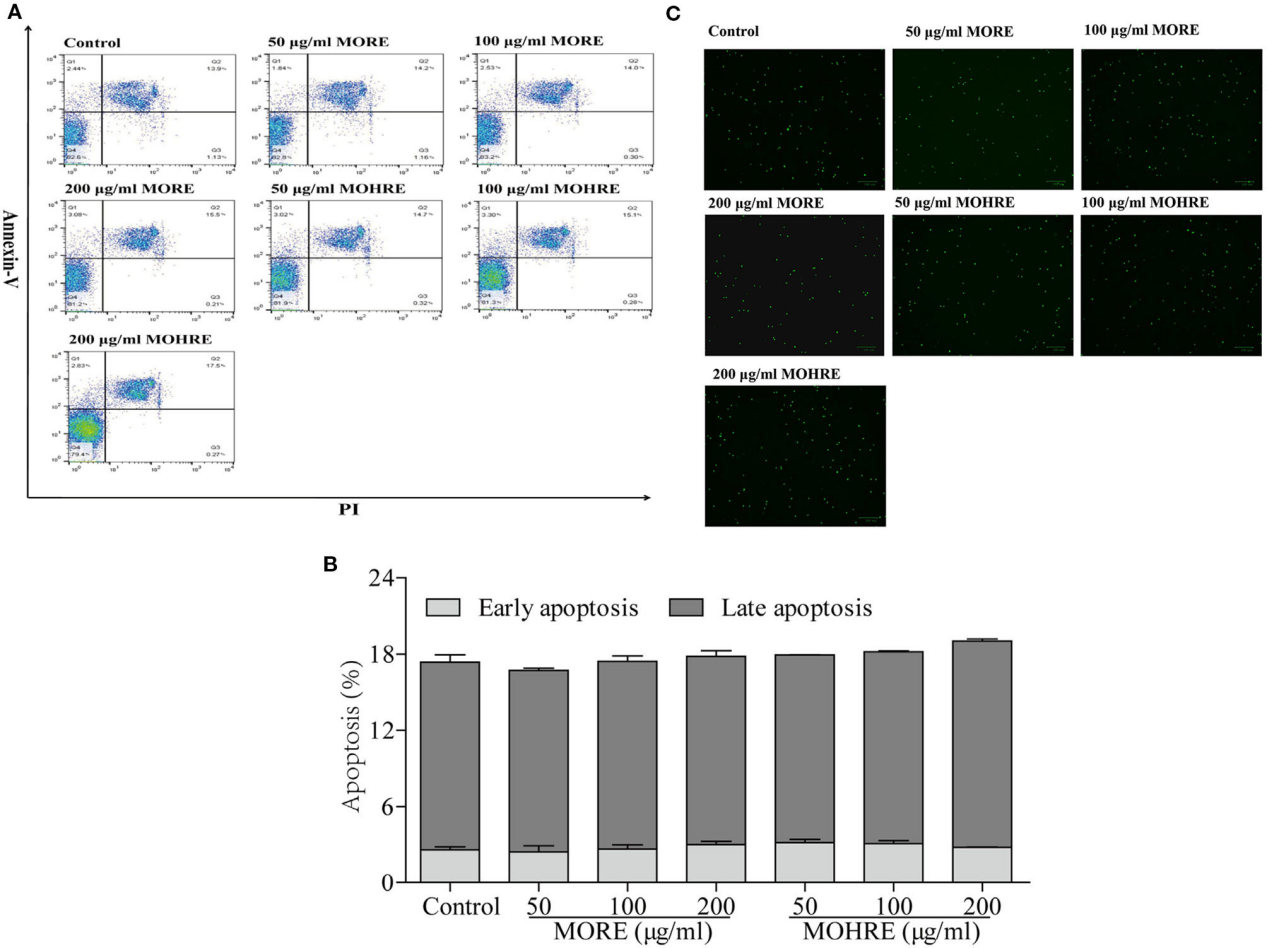
The apoptosis effect of root extract of Morinda officinalis (MORE) and hairy root extract of M. officinalis (MOHRE) on normal splenic lymphocytes. (A) Representative FACS picture in each group. (B) Apoptosis assay. (C) Representative images were obtained using a fluorescence microscope (100μm). All data are expressed as mean ± S.E.M of three independent experiments. *p < 0.05, **p < 0.01 vs. control group.

The authors apologize for this error and state that this does not change the scientific conclusions of the article in any way. The original article has been updated.

