# Supplementary material for: The Extracts of Morinda officinalis and Its Hairy Roots Attenuate Dextran Sodium Sulfate-Induced Chronic Ulcerative Colitis in Mice by Regulating Inflammation and Lymphocyte Apoptosis
Source: Front Immunol. 2017 Aug 2;8:905. doi: 10.3389/fimmu.2017.00905 (PMC5539173; doi:10.3389/fimmu.2017.00905)
Supplement: Supplementary file 1 [file Presentation_1.PDF]

Supplementary information1

The Extracts of *Morinda Officinalis* and Its Hairy-roots Attenuate DSS-induced Chronic Ulcerative Colitis in Mice by Regulating Inflammation and Lymphocyte Apoptosis

Jian Liang[1, a], Jiwang Liang[2, a], Hairong Hao[3, a], Huan Lin[1], Peng Wang[2], Yanfang Wu[1], Xiaoli Jiang[2], Chaodi Fu[2], Qian Li[1], Ping Ding[1], Huazhen Liu[4], Qingping Xiong[1], Xiaoping Lai[1], Lian Zhou[1*], Shamyuen Chan[2*], Shaozhen Hou[1*]

[1] Guangdong Provincial Key Laboratory of New Chinese Medicinals Development and Research, Guangzhou University of Chinese Medicine, Guangzhou, China

[2] Shenzhen Fan Mao Pharmaceutical Co., Limited, Shenzhen, China.

[3] Affiliated Huai'an Hospital of Xuzhou Medical University, Huai'an 223001, Jiangsu, PR China

[4] Guangdong Provincial Academy of Chinese Medical Sciences, and Guangdong Provincial Hospital of Chinese Medicine, Section of Immunology, Guangzhou, China

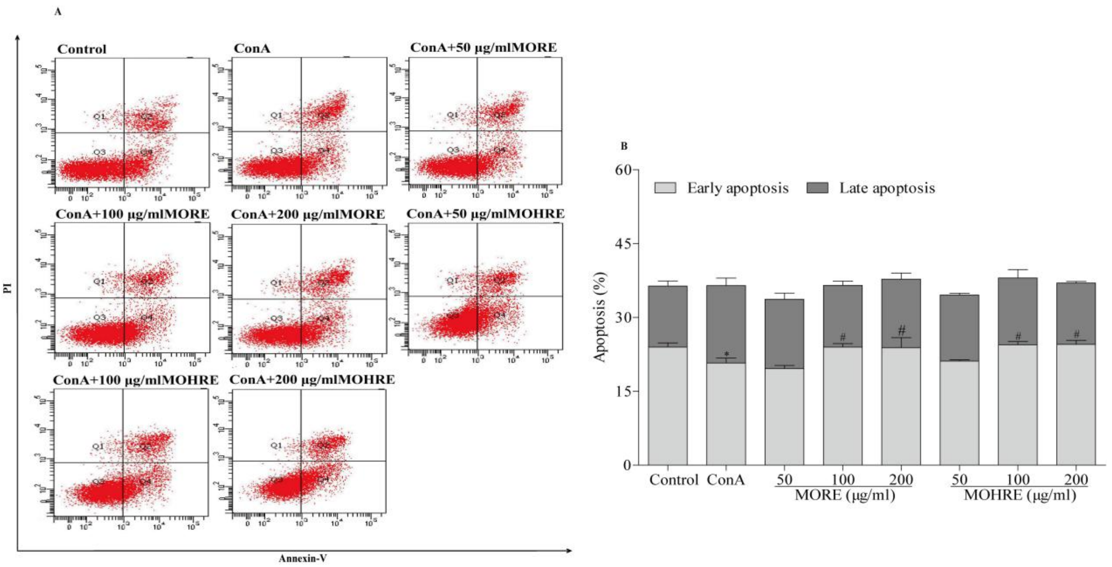

Supplementary Fig. 1. The apoptosis effect of MORE and MOHRE on spleen lymphocytes have been activated by ConA for 48h. (A) Representative FACS picture in each group. (B) Apoptosis assay. All data are expressed as means ± SEM of 3 independent experiments. *p<0.05. **p<0.01 vs. control group; #p<0.05. ##p<0.01 vs. DSS group.

---

[*]Corresponding author: E-mail address: zl@gzucm.edu.cn (Lian Zhou); samchan@phytogaa.com (Shamyuen Chan); hsz0214@gzucm.edu.cn (Shaozhen Hou).

[a] These authors contributed equally to this paper.

The apoptosis effect of MORE and MOHRE on spleen lymphocytes in the presence of ConA stimulation for 48h were provided in the Supplementary Fig. 1. The result showed that MORE and MOHRE could dose-dependently induce the early apoptosis of spleen lymphocytes, and MORE and MOHRE did not inflect the late apoptosis of spleen lymphocytes for 48h with ConA stimulation.
