# Supplementary material for: The Extracts of Morinda officinalis and Its Hairy Roots Attenuate Dextran Sodium Sulfate-Induced Chronic Ulcerative Colitis in Mice by Regulating Inflammation and Lymphocyte Apoptosis
Source: Front Immunol. 2017 Aug 2;8:905. doi: 10.3389/fimmu.2017.00905 (PMC5539173; doi:10.3389/fimmu.2017.00905)
Supplement: Supplementary file 2 [file Presentation_2.PDF]

Supplementary information2

The Extracts of *Morinda Officinalis* and Its Hairy-roots Attenuate DSS-induced Chronic Ulcerative Colitis in Mice by Regulating Inflammation and Lymphocyte Apoptosis

Jian Liang[1, a], Jiwang Liang[2, a], Hairong Hao[3, a], Huan Lin[1], Peng Wang[2], Yanfang Wu[1], Xiaoli Jiang[2], Chaodi Fu[2], Qian Li[1], Ping Ding[1], Huazhen Liu[4], Qingping Xiong[1], Xiaoping Lai[1], Lian Zhou[1*], Shamyuen Chan[2*], Shaozhen Hou[1*]

[1] Guangdong Provincial Key Laboratory of New Chinese Medicinals Development and Research, Guangzhou University of Chinese Medicine, Guangzhou, China
[2] Shenzhen Fan Mao Pharmaceutical Co., Limited, Shenzhen, China.
[3] Affiliated Huai'an Hospital of Xuzhou Medical University, Huai'an 223001, Jiangsu, PR China
[4] Guangdong Provincial Academy of Chinese Medical Sciences, and Guangdong Provincial Hospital of Chinese Medicine, Section of Immunology, Guangzhou, China

The apoptosis effects of MORE and MOHRE on lymphocytes with ConA stimulation for 24h. All original date were provided as follows:

MORE-L is MORE 50 μg/ml
MORE-M is MORE 100 μg/ml
MORE-H is MORE 200 μg/ml

MOHRE-L is MOHRE 50 μg/ml
MOHRE-M is MOHRE 100 μg/ml
MOHRE-H is MOHRE 200 μg/ml

[*] *Corresponding author:* E-mail address: *zl@gzucm.edu.cn (Lian Zhou); samchan@phytogaa.com (Shamyuen Chan); hsz0214@gzucm.edu.cn (Shaozhen Hou).*

[a] *These authors contributed equally to this paper.*

Batch Analysis Report

Run Date: 6/17/17 8:28 PM Experiment: Experiment_386

User ID: Administrator

Statistics Output: C:\Documents and Settings\Administrator\Desktop\LJ+CONA24\Batch_Analysis_17062017202747.csv

Worksheet PDF Output: C:\Documents and

Specimen_001

Tube	Status	Run Time
Negative_001	OK	6/17/17 8:28 PM
PI_002	OK	6/17/17 8:28 PM
FITC_003	OK	6/17/17 8:28 PM
Control_001	OK	6/17/17 8:28 PM
Control_002	OK	6/17/17 8:28 PM
Control_003	OK	6/17/17 8:28 PM
ConA_001	OK	6/17/17 8:28 PM
ConA_002	OK	6/17/17 8:28 PM
ConA_003	OK	6/17/17 8:28 PM
MORE-L_001	OK	6/17/17 8:28 PM
MORE-L_002	OK	6/17/17 8:28 PM
MORE-L_003	OK	6/17/17 8:28 PM
MORE-M_001	OK	6/17/17 8:28 PM
MORE-M_002	OK	6/17/17 8:28 PM
MORE-M_003	OK	6/17/17 8:28 PM
MORE-H_001	OK	6/17/17 8:28 PM
MORE-H_002	OK	6/17/17 8:28 PM
MORE-H_003	OK	6/17/17 8:28 PM
MOHRE-L_001	OK	6/17/17 8:28 PM
MOHRE-L_002	OK	6/17/17 8:28 PM
MOHRE-L_003	OK	6/17/17 8:28 PM
MOHRE-M_001	OK	6/17/17 8:28 PM
MOHRE-M_002	OK	6/17/17 8:28 PM
MOHRE-M_003	OK	6/17/17 8:28 PM
MOHRE-H_001	OK	6/17/17 8:28 PM
MOHRE-H_002	OK	6/17/17 8:28 PM
MOHRE-H_003	OK	6/17/17 8:28 PM

FACSDiva Version 6.1.3

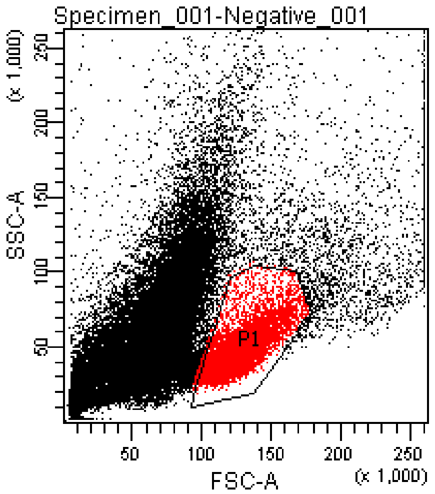

Tube: Negative_001			
Population	#Events	%Parent	%Total
All Events	49,609	####	100.0
P1	10,000	20.2	20.2
Q1	1	0.0	0.0
Q2	0	0.0	0.0
Q3	9,999	100.0	20.2
Q4	0	0.0	0.0

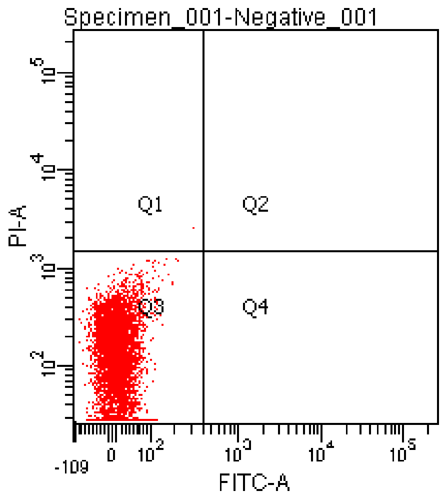

Experiment Name: Experiment_386
Specimen Name: Specimen_001
Tube Name: Negative_001
Record Date: Jun 17, 2017 7:10:51 PM
$OP: Administrator
GUID: c8227702-b8b7-46cc-819e-fca31f47e21d

Population	#Events	%Parent	FSC-A Mean	SSC-A Mean
All Events	49,609	####	82,100	61,509
P1	10,000	20.2	125,240	45,135
Q1	1	0.0	135,856	70,674
Q2	0	0.0	####	####
Q3	9,999	100.0	125,239	45,133
Q4	0	0.0	####	####

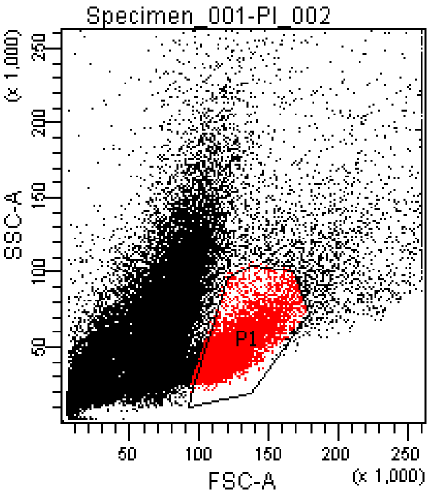

Tube: PI_002

Population	#Events	%Parent	%Total
All Events	50,877	####	100.0
P1	10,000	19.7	19.7
Q1	243	2.4	0.5
Q2	0	0.0	0.0
Q3	9,757	97.6	19.2
Q4	0	0.0	0.0

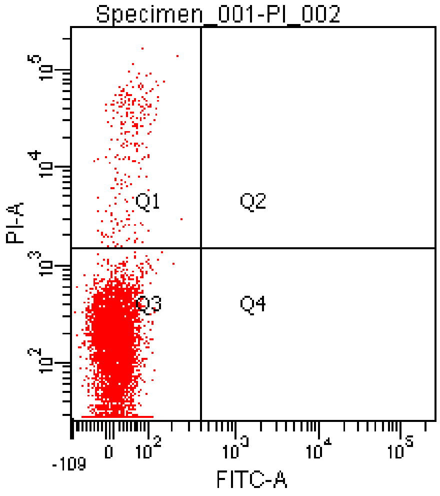

Experiment Name: Experiment_386
Specimen Name: Specimen_001
Tube Name: PI_002
Record Date: Jun 17, 2017 7:12:56 PM
$OP: Administrator
GUID: 606033b4-063c-475c-ac98-39b19c48fa9f

Population	#Events	%Parent	FSC-A Mean	SSC-A Mean
All Events	50,877	####	80,086	61,089
P1	10,000	19.7	124,299	46,809
Q1	243	2.4	132,504	76,368
Q2	0	0.0	####	####
Q3	9,757	97.6	124,095	46,073
Q4	0	0.0	####	####

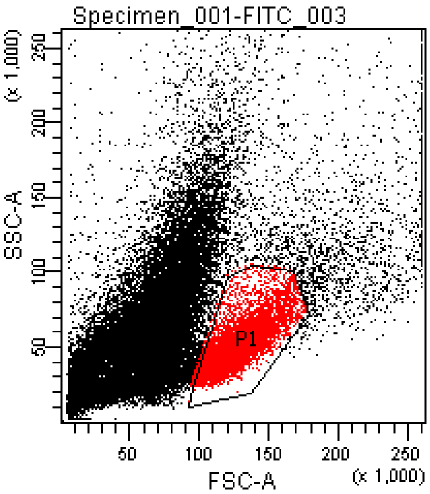

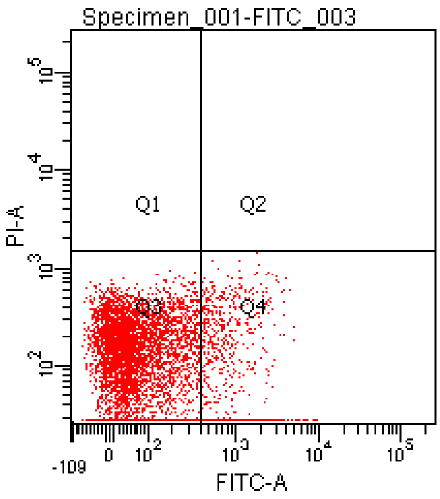

Tube: FITC_003

Population	#Events	%Parent	%Total
All Events	49,528	####	100.0
P1	10,000	20.2	20.2
Q1	1	0.0	0.0
Q2	0	0.0	0.0
Q3	8,794	87.9	17.8
Q4	1,205	12.0	2.4

Experiment Name: Experiment_386
Specimen Name: Specimen_001
Tube Name: FITC_003
Record Date: Jun 17, 2017 7:18:49 PM
$OP: Administrator
GUID: b2f1d976-a1f2-4d8e-966b-e27f0b05e1a4

Population	#Events	%Parent	FSC-A Mean	SSC-A Mean
All Events	49,528	####	80,371	61,158
P1	10,000	20.2	125,222	47,432
Q1	1	0.0	172,334	68,584
Q2	0	0.0	####	####
Q3	8,794	87.9	123,430	45,069
Q4	1,205	12.0	138,258	64,662

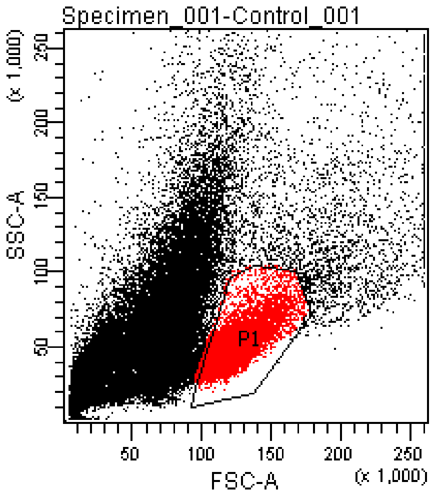

Tube: Control_001

Population	#Events	%Parent	%Total
All Events	50,803	####	100.0
P1	10,000	19.7	19.7
Q1	78	0.8	0.2
Q2	210	2.1	0.4
Q3	9,264	92.6	18.2
Q4	448	4.5	0.9

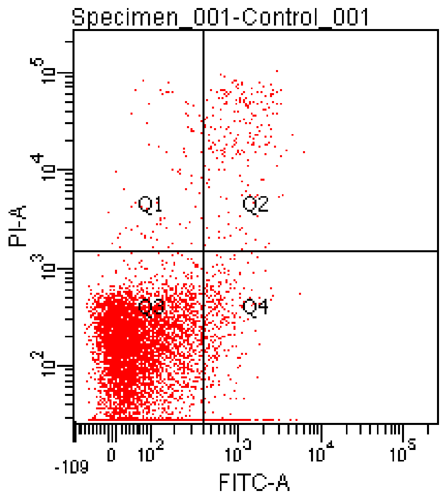

Experiment Name: Experiment_386
Specimen Name: Specimen_001
Tube Name: Control_001
Record Date: Jun 17, 2017 7:22:28 PM
$OP: Administrator
GUID: d5c1e33e-9f52-4654-b1cd-0d116ebfa1da

Population	#Events	%Parent	FSC-A Mean	SSC-A Mean
All Events	50,803	####	83,688	64,658
P1	10,000	19.7	124,382	48,877
Q1	78	0.8	132,550	63,193
Q2	210	2.1	133,731	78,697
Q3	9,264	92.6	123,330	47,217
Q4	448	4.5	140,337	66,725

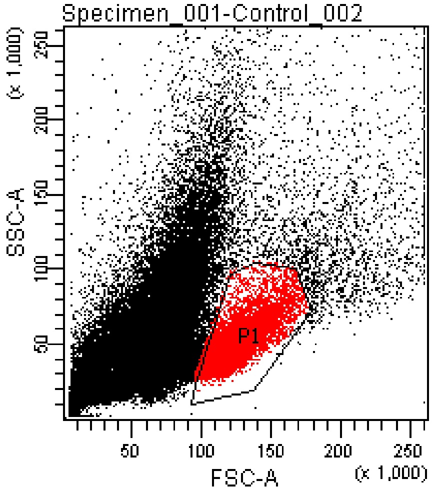

Tube: Control_002

Population	#Events	%Parent	%Total
All Events	51,522	####	100.0
P1	10,000	19.4	19.4
Q1	93	0.9	0.2
Q2	197	2.0	0.4
Q3	9,247	92.5	17.9
Q4	463	4.6	0.9

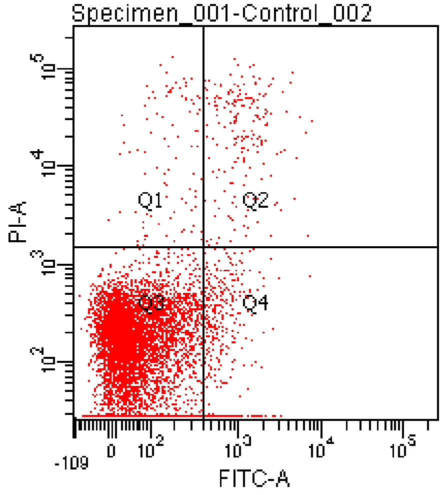

Experiment Name: Experiment_386
Specimen Name: Specimen_001
Tube Name: Control_002
Record Date: Jun 17, 2017 7:23:51 PM
$OP: Administrator
GUID: 576844e4-e6cc-430e-8a27-95d2ec084e3c

Population	#Events	%Parent	FSC-A Mean	SSC-A Mean
All Events	51,522	####	82,522	63,985
P1	10,000	19.4	125,053	48,887
Q1	93	0.9	130,831	63,320
Q2	197	2.0	134,603	78,069
Q3	9,247	92.5	124,034	47,258
Q4	463	4.6	140,160	66,118

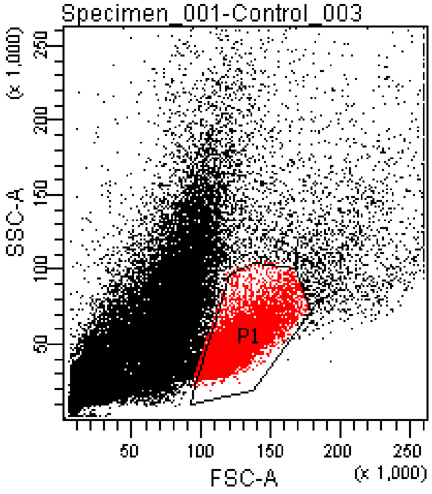

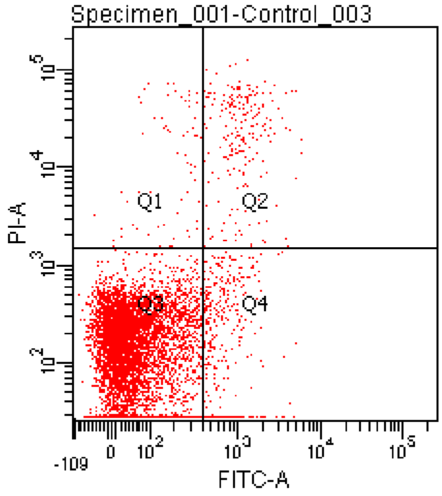

Tube: Control_003

Population	#Events	%Parent	%Total
All Events	47,796	####	100.0
P1	10,000	20.9	20.9
Q1	65	0.6	0.1
Q2	210	2.1	0.4
Q3	9,317	93.2	19.5
Q4	408	4.1	0.9

Experiment Name: Experiment_386
Specimen Name: Specimen_001
Tube Name: Control_003
Record Date: Jun 17, 2017 7:24:42 PM
$OP: Administrator
GUID: 6ae59970-dc8c-48e6-8ef6-9111b817f22d

Population	#Events	%Parent	FSC-A Mean	SSC-A Mean
All Events	47,796	####	84,537	64,454
P1	10,000	20.9	126,290	48,931
Q1	65	0.6	129,598	65,980
Q2	210	2.1	130,779	76,463
Q3	9,317	93.2	125,447	47,388
Q4	408	4.1	142,695	67,268

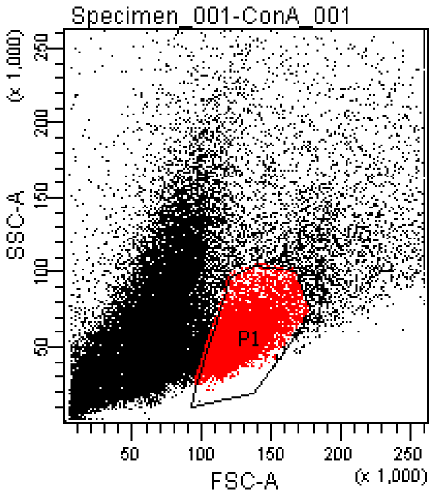

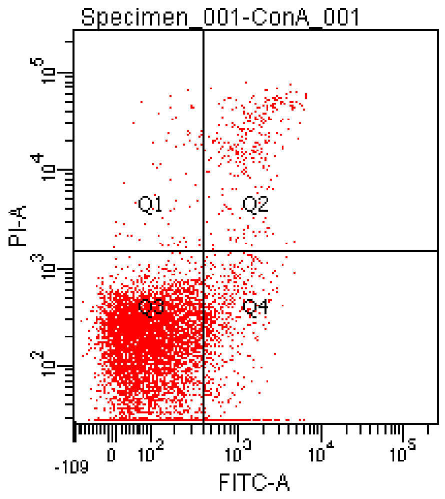

Tube: ConA_001

Population	#Events	%Parent	%Total
All Events	46,276	####	100.0
P1	10,000	21.6	21.6
Q1	68	0.7	0.1
Q2	302	3.0	0.7
Q3	8,813	88.1	19.0
Q4	817	8.2	1.8

Experiment Name: Experiment_386
Specimen Name: Specimen_001
Tube Name: ConA_001
Record Date: Jun 17, 2017 8:13:43 PM
$OP: Administrator
GUID: d8b7d53b-e590-4fe7-930a-cba09589ea53

Population	#Events	%Parent	FSC-A Mean	SSC-A Mean
All Events	46,276	####	88,904	70,225
P1	10,000	21.6	127,539	55,408
Q1	68	0.7	133,024	67,519
Q2	302	3.0	132,918	77,417
Q3	8,813	88.1	126,710	53,674
Q4	817	8.2	134,040	64,975

FACSDiva Version 6.1.3

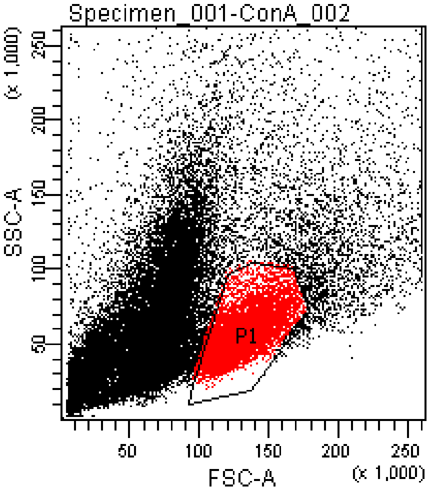

Tube: ConA_002

Population	#Events	%Parent	%Total
All Events	42,961	####	100.0
P1	10,000	23.3	23.3
Q1	77	0.8	0.2
Q2	250	2.5	0.6
Q3	8,901	89.0	20.7
Q4	772	7.7	1.8

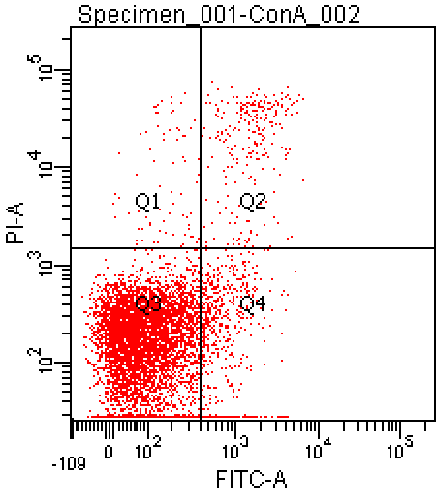

Experiment Name: Experiment_386
Specimen Name: Specimen_001
Tube Name: ConA_002
Record Date: Jun 17, 2017 8:17:37 PM
$OP: Administrator
GUID: 0181f391-0338-4973-8c41-aec6d74a9ee2

Population	#Events	%Parent	FSC-A Mean	SSC-A Mean
All Events	42,961	####	90,644	69,701
P1	10,000	23.3	128,922	52,939
Q1	77	0.8	133,508	65,981
Q2	250	2.5	130,714	75,575
Q3	8,901	89.0	128,124	51,256
Q4	772	7.7	137,077	63,710

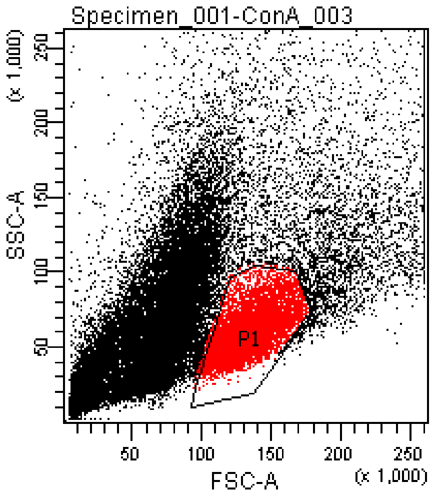

Tube: ConA_003

Population	#Events	%Parent	%Total
All Events	44,515	####	100.0
P1	10,000	22.5	22.5
Q1	72	0.7	0.2
Q2	302	3.0	0.7
Q3	8,884	88.8	20.0
Q4	742	7.4	1.7

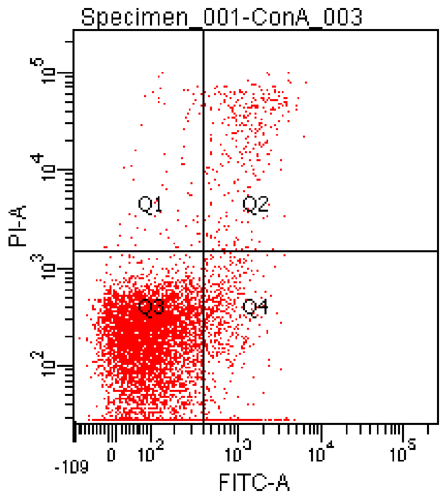

Experiment Name: Experiment_386
Specimen Name: Specimen_001
Tube Name: ConA_003
Record Date: Jun 17, 2017 7:28:13 PM
$OP: Administrator
GUID: c0dab0c7-6886-4e13-b4f4-ea9f49a23d4c

Population	#Events	%Parent	FSC-A Mean	SSC-A Mean
All Events	44,515	####	97,944	75,791
P1	10,000	22.5	133,341	57,093
Q1	72	0.7	136,457	67,996
Q2	302	3.0	131,057	77,030
Q3	8,884	88.8	132,864	55,611
Q4	742	7.4	139,680	65,658

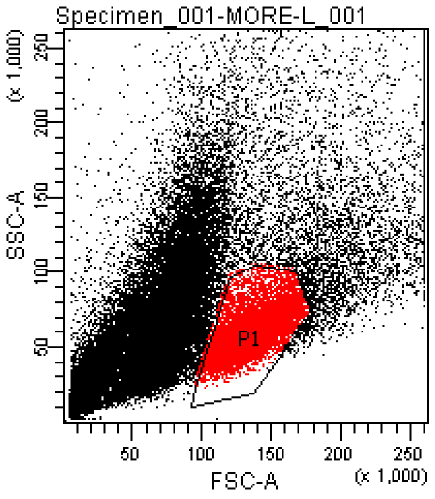

Tube: MORE-L_001

Population	#Events	%Parent	%Total
All Events	51,934	####	100.0
P1	10,000	19.3	19.3
Q1	70	0.7	0.1
Q2	425	4.2	0.8
Q3	8,299	83.0	16.0
Q4	1,206	12.1	2.3

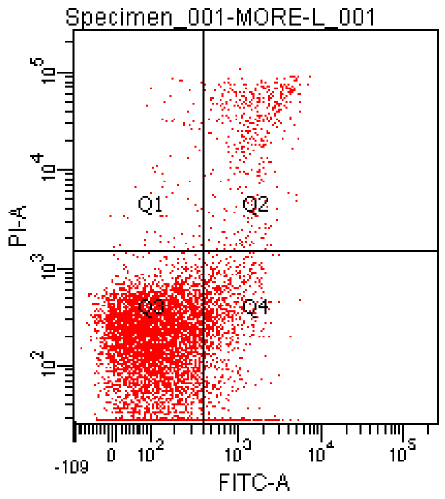

Experiment Name: Experiment_386
Specimen Name: Specimen_001
Tube Name: MORE-L_001
Record Date: Jun 17, 2017 7:29:27 PM
$OP: Administrator
GUID: f4fb36e4-a050-47ff-ad41-8b32ae04081f

Population	#Events	%Parent	FSC-A Mean	SSC-A Mean
All Events	51,934	####	95,400	76,296
P1	10,000	19.3	134,211	57,656
Q1	70	0.7	135,782	65,450
Q2	425	4.2	130,935	77,992
Q3	8,299	83.0	133,646	55,562
Q4	1,206	12.1	139,159	64,445

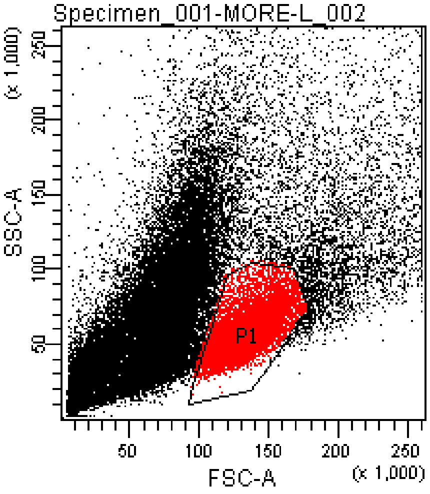

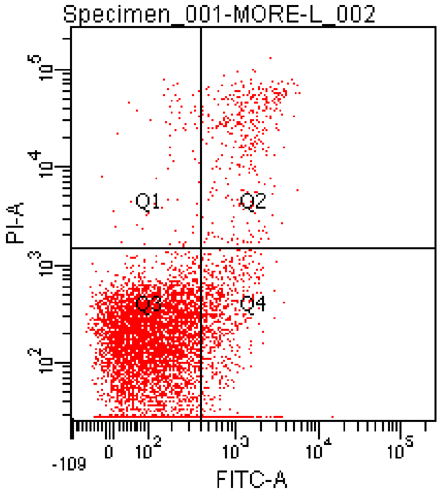

Tube: MORE-L_002

Population	#Events	%Parent	%Total
All Events	52,747	####	100.0
P1	10,000	19.0	19.0
Q1	76	0.8	0.1
Q2	363	3.6	0.7
Q3	8,538	85.4	16.2
Q4	1,023	10.2	1.9

Experiment Name: Experiment_386
Specimen Name: Specimen_001
Tube Name: MORE-L_002
Record Date: Jun 17, 2017 7:30:29 PM
$OP: Administrator
GUID: b9498889-5485-4c69-bebc-ed1982dea95d

Population	#Events	%Parent	FSC-A Mean	SSC-A Mean
All Events	52,747	####	95,778	77,265
P1	10,000	19.0	133,833	56,547
Q1	76	0.8	132,356	66,627
Q2	363	3.6	130,580	77,164
Q3	8,538	85.4	133,247	54,600
Q4	1,023	10.2	139,986	64,740

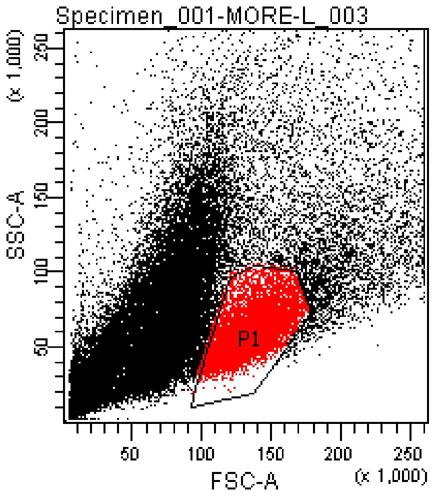

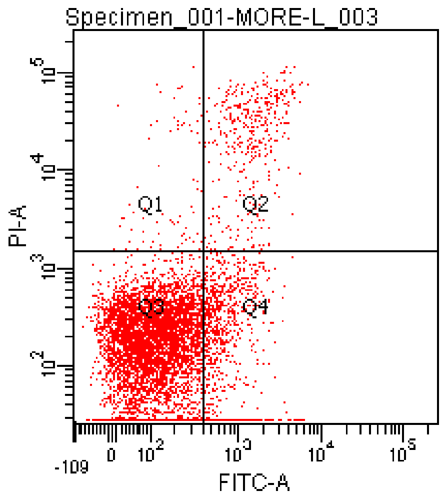

Tube: MORE-L_003

Population	#Events	%Parent	%Total
All Events	52,115	####	100.0
P1	10,000	19.2	19.2
Q1	72	0.7	0.1
Q2	396	4.0	0.8
Q3	8,328	83.3	16.0
Q4	1,204	12.0	2.3

Experiment Name: Experiment_386
Specimen Name: Specimen_001
Tube Name: MORE-L_003
Record Date: Jun 17, 2017 7:31:37 PM
$OP: Administrator
GUID: 76d39abc-33e3-4978-b586-31f6ae2ffb9f

Population	#Events	%Parent	FSC-A Mean	SSC-A Mean
All Events	52,115	####	96,073	77,684
P1	10,000	19.2	134,091	57,940
Q1	72	0.7	132,899	65,136
Q2	396	4.0	133,063	78,663
Q3	8,328	83.3	133,331	55,791
Q4	1,204	12.0	139,756	65,562

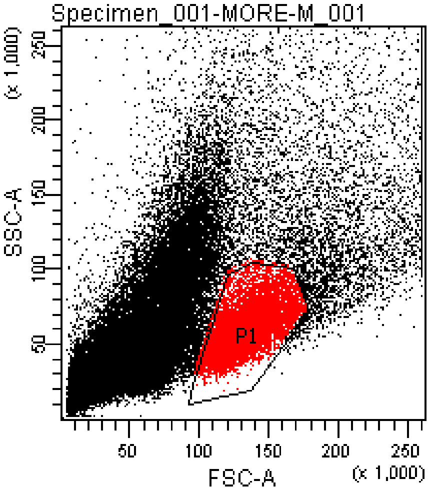

Tube: MORE-M_001			
Population	#Events	%Parent	%Total
All Events	55,143	####	100.0
P1	10,000	18.1	18.1
Q1	81	0.8	0.1
Q2	478	4.8	0.9
Q3	8,276	82.8	15.0
Q4	1,165	11.6	2.1

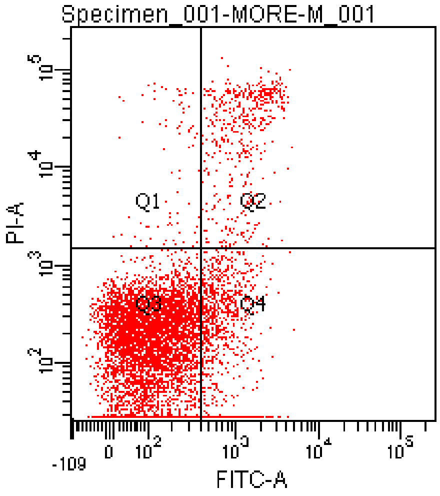

Experiment Name: Experiment_386
Specimen Name: Specimen_001
Tube Name: MORE-M_001
Record Date: Jun 17, 2017 7:32:50 PM
$OP: Administrator
GUID: c2771bbc-edcf-49c7-b9bb-75bd59cb2112

Population	#Events	%Parent	FSC-A Mean	SSC-A Mean
All Events	55,143	####	91,969	74,524
P1	10,000	18.1	133,694	58,189
Q1	81	0.8	127,854	64,005
Q2	478	4.8	131,231	75,508
Q3	8,276	82.8	133,041	55,961
Q4	1,165	11.6	139,749	66,500

FACSDiva Version 6.1.3

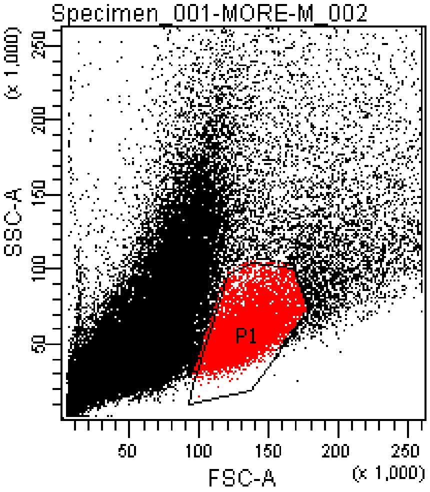

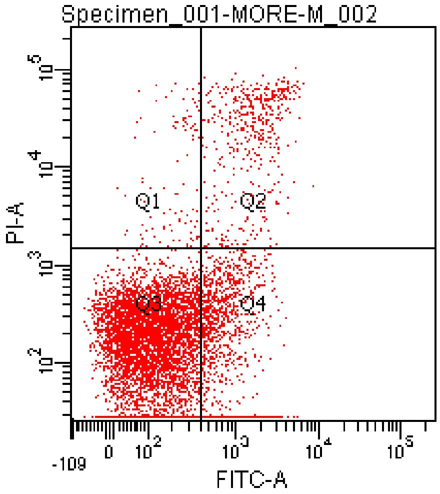

Tube: MORE-M_002

Population	#Events	%Parent	%Total
All Events	61,931	####	100.0
P1	10,000	16.1	16.1
Q1	98	1.0	0.2
Q2	466	4.7	0.8
Q3	7,987	79.9	12.9
Q4	1,449	14.5	2.3

Experiment Name: Experiment_386
Specimen Name: Specimen_001
Tube Name: MORE-M_002
Record Date: Jun 17, 2017 7:34:46 PM
$OP: Administrator
GUID: 4934723d-9aa9-43d2-8837-986be4274ac3

Population	#Events	%Parent	FSC-A Mean	SSC-A Mean
All Events	61,931	####	93,092	78,442
P1	10,000	16.1	134,038	57,489
Q1	98	1.0	133,681	64,868
Q2	466	4.7	131,267	77,089
Q3	7,987	79.9	133,457	55,128
Q4	1,449	14.5	138,151	63,705

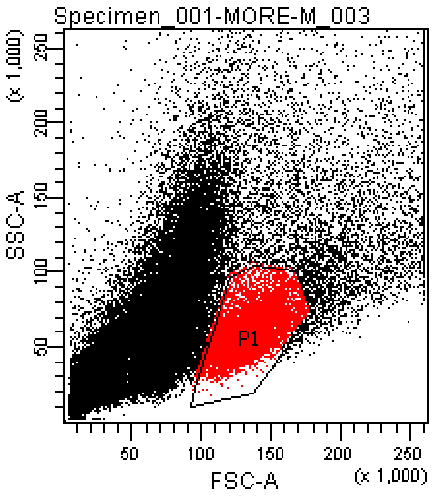

Tube: MORE-M_003

Population	#Events	%Parent	%Total
All Events	58,904	####	100.0
P1	10,000	17.0	17.0
Q1	86	0.9	0.1
Q2	462	4.6	0.8
Q3	8,149	81.5	13.8
Q4	1,303	13.0	2.2

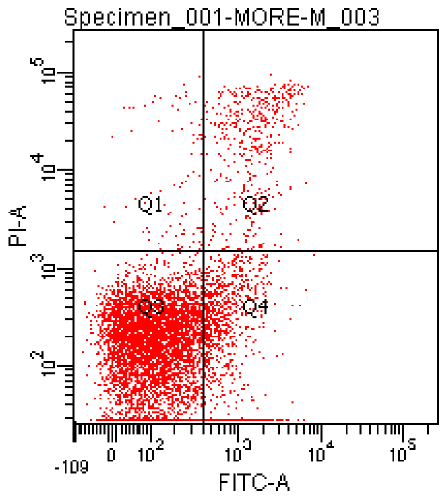

Experiment Name: Experiment_386
Specimen Name: Specimen_001
Tube Name: MORE-M_003
Record Date: Jun 17, 2017 7:35:58 PM
$OP: Administrator
GUID: c1890a12-9277-4b66-a3f1-bfa013229619

Population	#Events	%Parent	FSC-A Mean	SSC-A Mean
All Events	58,904	####	93,475	77,241
P1	10,000	17.0	133,943	57,777
Q1	86	0.9	133,886	68,869
Q2	462	4.6	130,673	77,468
Q3	8,149	81.5	133,449	55,588
Q4	1,303	13.0	138,193	63,750

FACSDiva Version 6.1.3

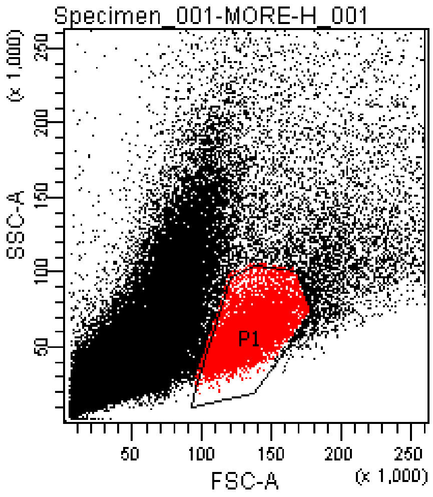

Tube: MORE-H_001			
Population	#Events	%Parent	%Total
All Events	61,289	####	100.0
P1	10,000	16.3	16.3
Q1	65	0.6	0.1
Q2	426	4.3	0.7
Q3	8,254	82.5	13.5
Q4	1,255	12.6	2.0

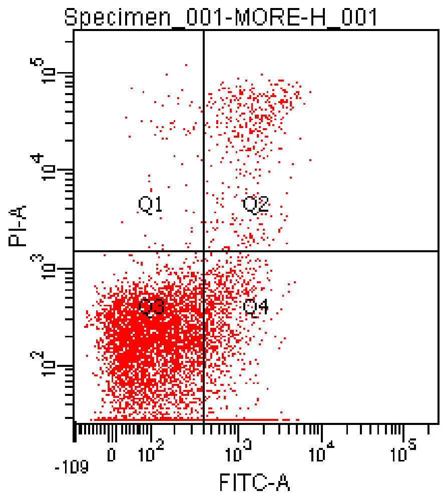

Experiment Name: Experiment_386
Specimen Name: Specimen_001
Tube Name: MORE-H_001
Record Date: Jun 17, 2017 7:37:21 PM
$OP: Administrator
GUID: c20c18ea-25d9-4157-95d5-6f916d732bfc

Population	#Events	%Parent	FSC-A Mean	SSC-A Mean
All Events	61,289	####	91,666	76,775
P1	10,000	16.3	132,815	57,665
Q1	65	0.6	128,985	66,078
Q2	426	4.3	132,592	79,252
Q3	8,254	82.5	132,342	55,495
Q4	1,255	12.6	136,201	64,176

FACSDiva Version 6.1.3

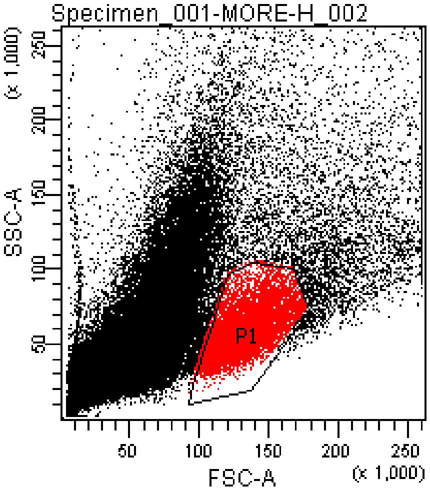

Tube: MORE-H_002			
Population	#Events	%Parent	%Total
All Events	62,537	####	100.0
P1	10,000	16.0	16.0
Q1	73	0.7	0.1
Q2	450	4.5	0.7
Q3	7,894	78.9	12.6
Q4	1,583	15.8	2.5

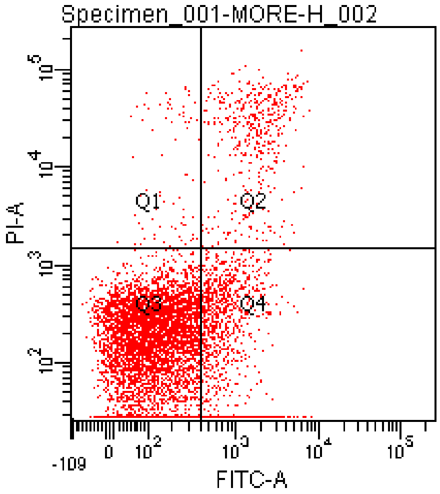

Experiment Name: Experiment_386
Specimen Name: Specimen_001
Tube Name: MORE-H_002
Record Date: Jun 17, 2017 7:40:54 PM
$OP: Administrator
GUID: c176444a-4a5a-4f82-b16d-4f725cd35342

Population	#Events	%Parent	FSC-A Mean	SSC-A Mean
All Events	62,537	####	90,619	76,561
P1	10,000	16.0	132,477	56,321
Q1	73	0.7	128,729	59,672
Q2	450	4.5	132,018	77,115
Q3	7,894	78.9	131,517	53,891
Q4	1,583	15.8	137,570	62,372

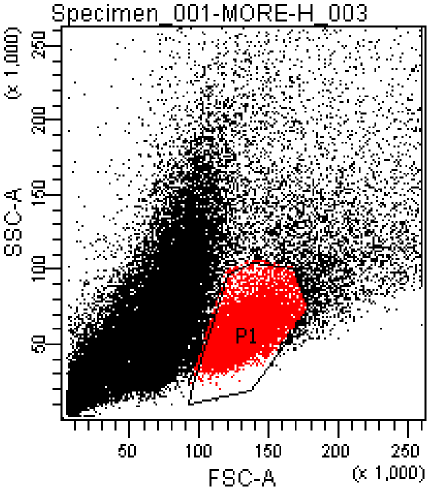

Tube: MORE-H_003			
Population	#Events	%Parent	%Total
All Events	53,721	####	100.0
P1	10,000	18.6	18.6
Q1	76	0.8	0.1
Q2	424	4.2	0.8
Q3	8,169	81.7	15.2
Q4	1,331	13.3	2.5

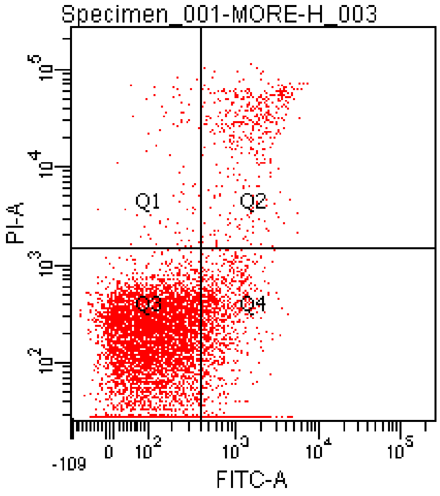

Experiment Name: Experiment_386
Specimen Name: Specimen_001
Tube Name: MORE-H_003
Record Date: Jun 17, 2017 7:39:30 PM
$OP: Administrator
GUID: 94309e16-e698-4d57-b6e0-b7f8fe1c1fba

Population	#Events	%Parent	FSC-A Mean	SSC-A Mean
All Events	53,721	####	94,468	76,315
P1	10,000	18.6	132,887	57,307
Q1	76	0.8	134,376	69,097
Q2	424	4.2	130,999	76,514
Q3	8,169	81.7	132,239	55,208
Q4	1,331	13.3	137,380	63,398

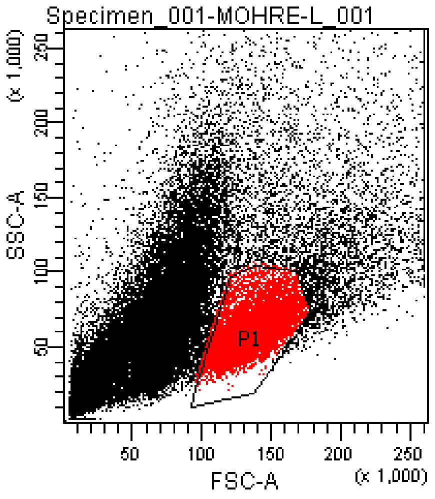

Tube: MOHRE-L_001

Population	#Events	%Parent	%Total
All Events	48,996	####	100.0
P1	10,000	20.4	20.4
Q1	92	0.9	0.2
Q2	431	4.3	0.9
Q3	8,327	83.3	17.0
Q4	1,150	11.5	2.3

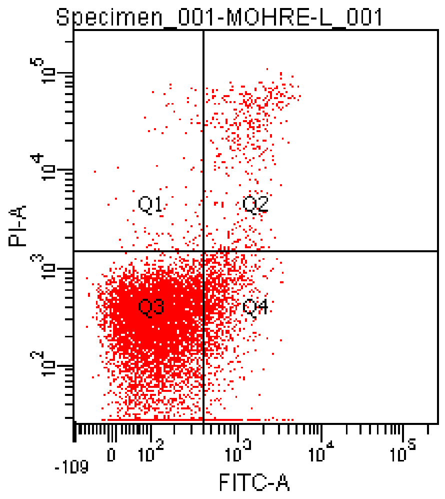

Experiment Name: Experiment_386
Specimen Name: Specimen_001
Tube Name: MOHRE-L_001
Record Date: Jun 17, 2017 7:41:58 PM
$OP: Administrator
GUID: 74e4f1c0-44ec-47ad-bc5e-bdd607777283

Population	#Events	%Parent	FSC-A Mean	SSC-A Mean
All Events	48,996	####	94,237	74,410
P1	10,000	20.4	134,351	58,380
Q1	92	0.9	136,791	66,585
Q2	431	4.3	131,402	76,437
Q3	8,327	83.3	133,691	56,278
Q4	1,150	11.5	140,041	66,177

FACSDiva Version 6.1.3

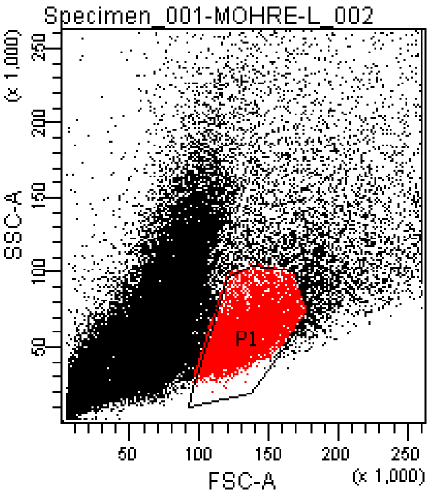

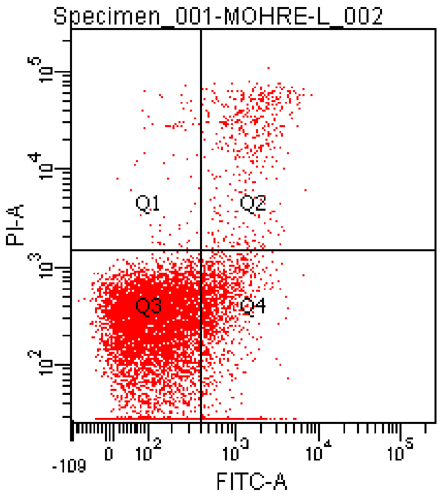

Tube: MOHRE-L_002

Population	#Events	%Parent	%Total
All Events	54,047	####	100.0
P1	10,000	18.5	18.5
Q1	64	0.6	0.1
Q2	451	4.5	0.8
Q3	8,126	81.3	15.0
Q4	1,359	13.6	2.5

Experiment Name: Experiment_386
Specimen Name: Specimen_001
Tube Name: MOHRE-L_002
Record Date: Jun 17, 2017 7:43:03 PM
$OP: Administrator
GUID: 11064f75-ecec-4abd-b305-b14611af8a6c

Population	#Events	%Parent	FSC-A Mean	SSC-A Mean
All Events	54,047	####	93,381	75,503
P1	10,000	18.5	132,759	57,277
Q1	64	0.6	132,735	64,765
Q2	451	4.5	132,972	77,740
Q3	8,126	81.3	131,960	55,124
Q4	1,359	13.6	137,466	63,005

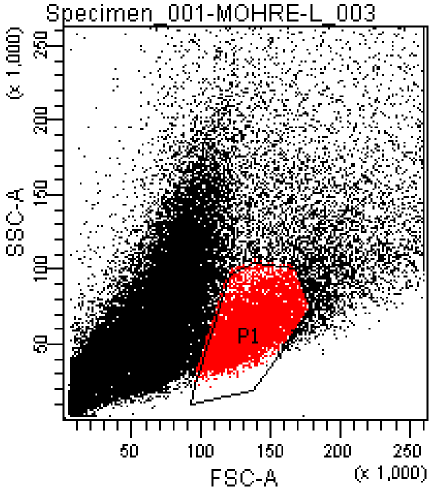

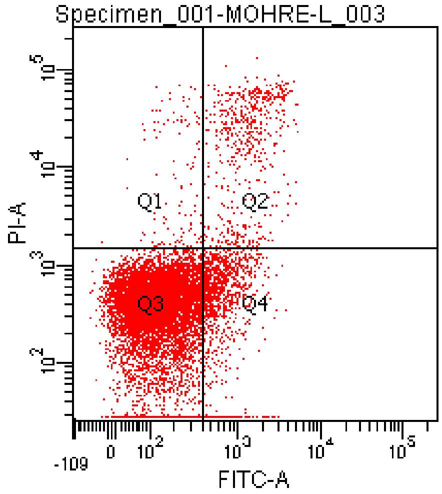

Tube: MOHRE-L_003

Population	#Events	%Parent	%Total
All Events	57,902	####	100.0
P1	10,000	17.3	17.3
Q1	111	1.1	0.2
Q2	537	5.4	0.9
Q3	8,251	82.5	14.2
Q4	1,101	11.0	1.9

Experiment Name: Experiment_386
Specimen Name: Specimen_001
Tube Name: MOHRE-L_003
Record Date: Jun 17, 2017 7:47:55 PM
$OP: Administrator
GUID: 8f758638-9f71-46ee-a208-6f3e52d771fb

Population	#Events	%Parent	FSC-A Mean	SSC-A Mean
All Events	57,902	####	92,145	75,641
P1	10,000	17.3	133,153	57,972
Q1	111	1.1	133,673	67,474
Q2	537	5.4	133,958	78,060
Q3	8,251	82.5	132,504	55,676
Q4	1,101	11.0	137,570	64,419

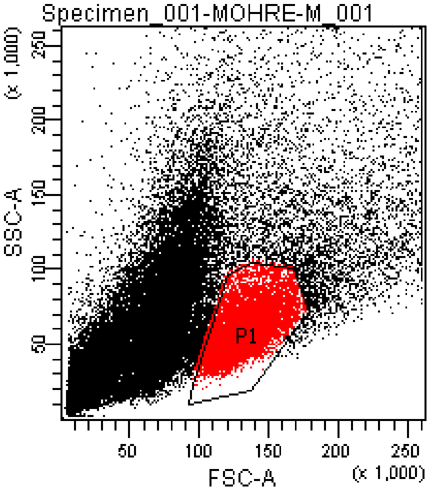

Tube: MOHRE-M_001

Population	#Events	%Parent	%Total
All Events	54,178	####	100.0
P1	10,000	18.5	18.5
Q1	69	0.7	0.1
Q2	474	4.7	0.9
Q3	8,274	82.7	15.3
Q4	1,183	11.8	2.2

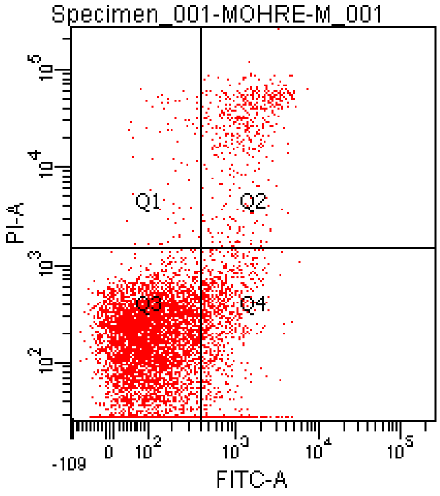

Experiment Name: Experiment_386
Specimen Name: Specimen_001
Tube Name: MOHRE-M_001
Record Date: Jun 17, 2017 7:56:25 PM
$OP: Administrator
GUID: f95b3864-aa27-43d4-954f-6f6993061818

Population	#Events	%Parent	FSC-A Mean	SSC-A Mean
All Events	54,178	####	87,691	70,904
P1	10,000	18.5	131,269	58,099
Q1	69	0.7	125,421	64,764
Q2	474	4.7	131,794	75,954
Q3	8,274	82.7	130,391	55,851
Q4	1,183	11.8	137,544	66,274

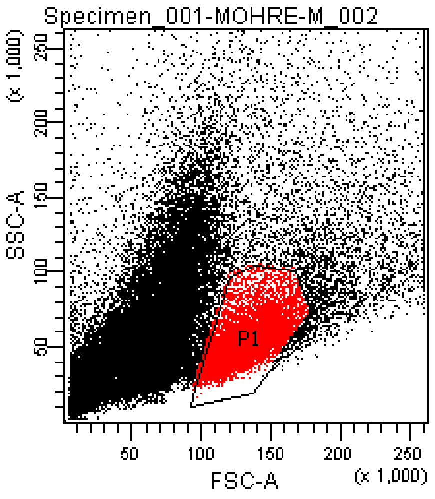

Tube: MOHRE-M_002

Population	#Events	%Parent	%Total
All Events	52,453	####	100.0
P1	10,000	19.1	19.1
Q1	76	0.8	0.1
Q2	429	4.3	0.8
Q3	8,161	81.6	15.6
Q4	1,334	13.3	2.5

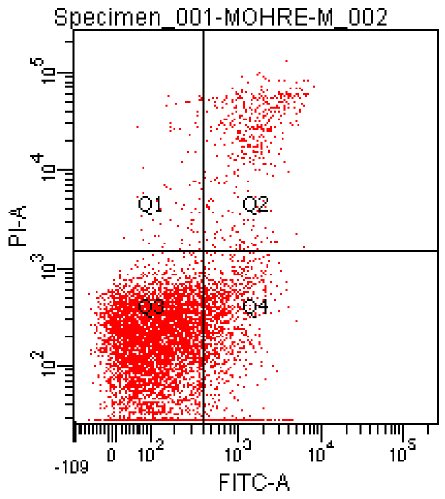

Experiment Name: Experiment_386
Specimen Name: Specimen_001
Tube Name: MOHRE-M_002
Record Date: Jun 17, 2017 7:57:33 PM
$OP: Administrator
GUID: a8fad7f7-5907-4c5e-985a-cf9126bea440

Population	#Events	%Parent	FSC-A Mean	SSC-A Mean
All Events	52,453	####	90,225	72,313
P1	10,000	19.1	130,465	51,809
Q1	76	0.8	132,678	64,480
Q2	429	4.3	130,914	74,293
Q3	8,161	81.6	129,547	49,221
Q4	1,334	13.3	135,816	59,690

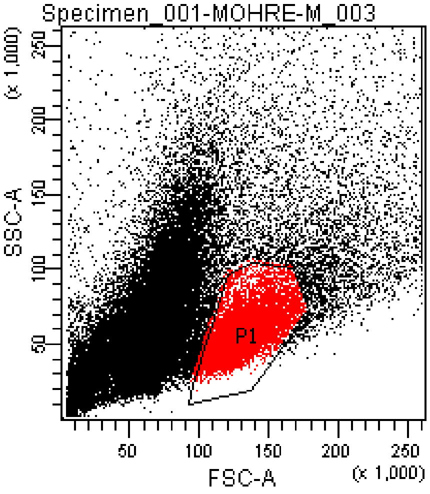

Tube: MOHRE-M_003

Population	#Events	%Parent	%Total
All Events	50,755	####	100.0
P1	10,000	19.7	19.7
Q1	89	0.9	0.2
Q2	378	3.8	0.7
Q3	8,401	84.0	16.6
Q4	1,132	11.3	2.2

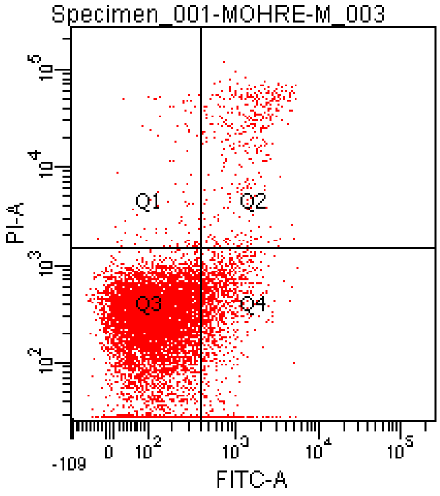

Experiment Name:	Experiment_386
Specimen Name:	Specimen_001
Tube Name:	MOHRE-M_003
Record Date:	Jun 17, 2017 7:58:34 PM
$OP:	Administrator
GUID:	76bb4064-dc6a-4b24-84a4-fd8250c18d90

Population	#Events	%Parent	FSC-A Mean	SSC-A Mean
All Events	50,755	####	89,406	71,682
P1	10,000	19.7	131,267	55,821
Q1	89	0.9	130,965	64,300
Q2	378	3.8	133,927	77,054
Q3	8,401	84.0	130,358	53,697
Q4	1,132	11.3	137,148	63,821

FACSDiva Version 6.1.3

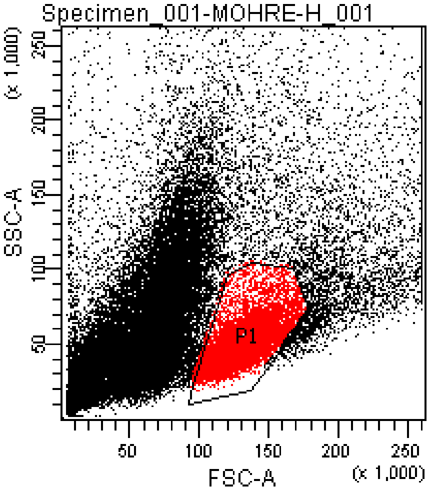

Tube: MOHRE-H_001			
Population	#Events	%Parent	%Total
All Events	52,043	####	100.0
P1	10,000	19.2	19.2
Q1	70	0.7	0.1
Q2	408	4.1	0.8
Q3	8,259	82.6	15.9
Q4	1,263	12.6	2.4

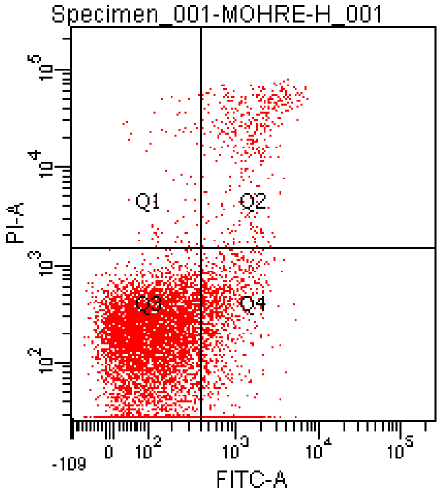

Experiment Name: Experiment_386
Specimen Name: Specimen_001
Tube Name: MOHRE-H_001
Record Date: Jun 17, 2017 7:59:45 PM
$OP: Administrator
GUID: 1ff19c07-3df3-4b66-844e-12cfc23f0541

Population	#Events	%Parent	FSC-A Mean	SSC-A Mean
All Events	52,043	####	89,287	70,748
P1	10,000	19.2	130,011	48,481
Q1	70	0.7	132,814	62,895
Q2	408	4.1	133,457	75,327
Q3	8,259	82.6	128,866	45,752
Q4	1,263	12.6	136,233	56,849

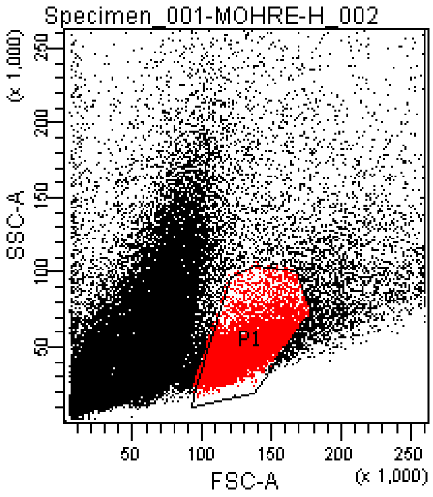

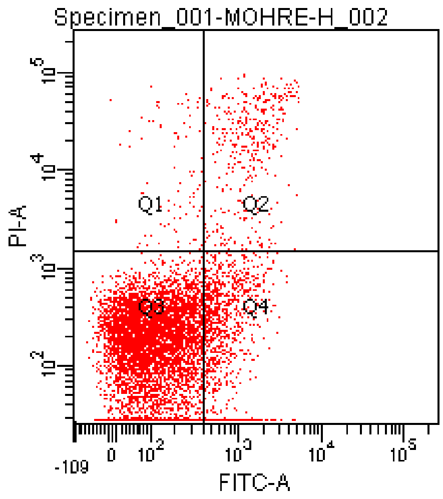

Tube: MOHRE-H_002

Population	#Events	%Parent	%Total
All Events	52,386	####	100.0
P1	10,000	19.1	19.1
Q1	87	0.9	0.2
Q2	381	3.8	0.7
Q3	8,326	83.3	15.9
Q4	1,206	12.1	2.3

Experiment Name: Experiment_386
Specimen Name: Specimen_001
Tube Name: MOHRE-H_002
Record Date: Jun 17, 2017 8:07:03 PM
$OP: Administrator
GUID: bd4c7bd6-8cb2-458c-ad9e-a23c46efc37f

Population	#Events	%Parent	FSC-A Mean	SSC-A Mean
All Events	52,386	####	85,165	69,075
P1	10,000	19.1	128,012	45,979
Q1	87	0.9	129,253	59,207
Q2	381	3.8	134,666	72,496
Q3	8,326	83.3	126,794	43,403
Q4	1,206	12.1	134,226	54,430

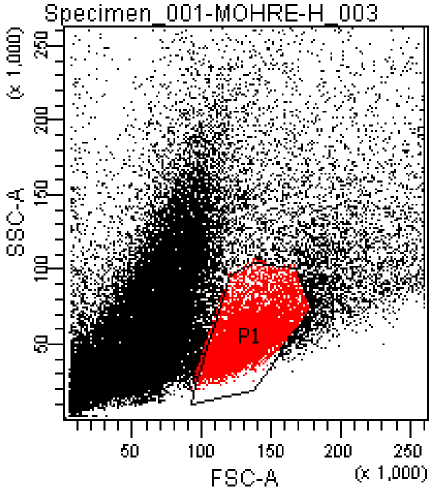

Tube: MOHRE-H_003			
Population	#Events	%Parent	%Total
All Events	56,395	####	100.0
P1	10,000	17.7	17.7
Q1	140	1.4	0.2
Q2	412	4.1	0.7
Q3	8,272	82.7	14.7
Q4	1,176	11.8	2.1

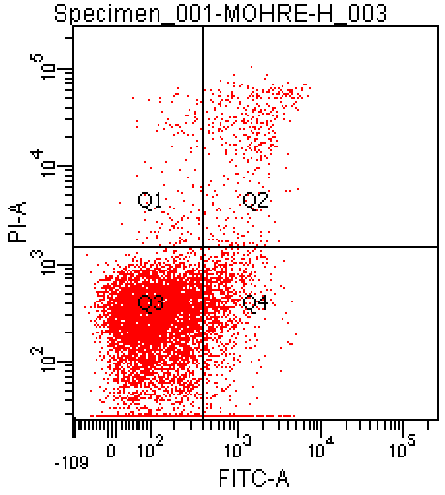

Experiment Name: Experiment_386
Specimen Name: Specimen_001
Tube Name: MOHRE-H_003
Record Date: Jun 17, 2017 8:09:45 PM
$OP: Administrator
GUID: cb791668-9625-4e97-a8b8-c452cedc6162

Population	#Events	%Parent	FSC-A Mean	SSC-A Mean
All Events	56,395	####	86,629	71,306
P1	10,000	17.7	129,616	51,459
Q1	140	1.4	127,894	62,357
Q2	412	4.1	132,307	75,774
Q3	8,272	82.7	128,779	48,974
Q4	1,176	11.8	134,769	59,123
