# Supplementary material for: The Extracts of Morinda officinalis and Its Hairy Roots Attenuate Dextran Sodium Sulfate-Induced Chronic Ulcerative Colitis in Mice by Regulating Inflammation and Lymphocyte Apoptosis
Source: Front Immunol. 2017 Aug 2;8:905. doi: 10.3389/fimmu.2017.00905 (PMC5539173; doi:10.3389/fimmu.2017.00905)
Supplement: Supplementary file 3 [file Presentation_3.PDF]

Supplementary information3

The Extracts of *Morinda Officinalis* and Its Hairy-roots Attenuate DSS-induced Chronic Ulcerative Colitis in Mice by Regulating Inflammation and Lymphocyte Apoptosis

Jian Liang[1, a], Jiwang Liang[2, a], Hairong Hao[3, a], Huan Lin[1], Peng Wang[2], Yanfang Wu[1], Xiaoli Jiang[2], Chaodi Fu[2], Qian Li[1], Ping Ding[1], Huazhen Liu[4], Qingping Xiong[1], Xiaoping Lai[1], Lian Zhou[1*], Shamyuen Chan[2*], Shaozhen Hou[1*]

[1] Guangdong Provincial Key Laboratory of New Chinese Medicinals Development and Research, Guangzhou University of Chinese Medicine, Guangzhou, China

[2] Shenzhen Fan Mao Pharmaceutical Co., Limited, Shenzhen, China.

[3] Affiliated Huai'an Hospital of Xuzhou Medical University, Huai'an 223001, Jiangsu, PR China

[4] Guangdong Provincial Academy of Chinese Medical Sciences, and Guangdong Provincial Hospital of Chinese Medicine, Section of Immunology, Guangzhou, China

The result came from the original manuscript and reanalyzed by Flowjo software. All results showed as follows:

[*]Corresponding author: E-mail address: zl@gzucm.edu.cn (Lian Zhou); samchan@phytogaa.com (Shamyuen Chan); hsz0214@gzucm.edu.cn (Shaozhen Hou).

[a] These authors contributed equally to this paper.

Control Group

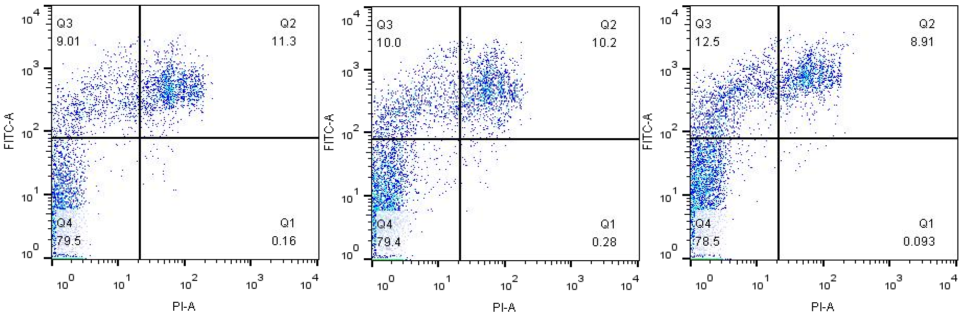

ConA Group

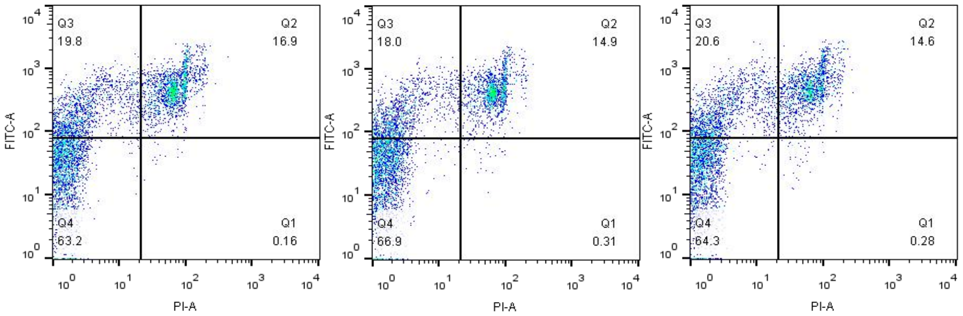

MORE 50 (μg/ml) Group

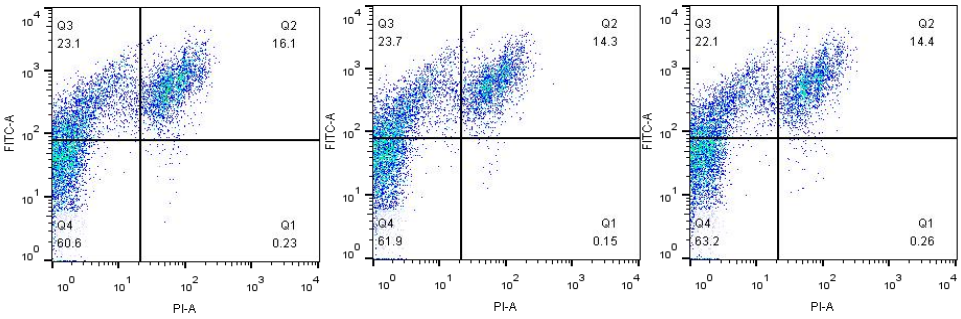

MORE 100 (μg/ml) Group

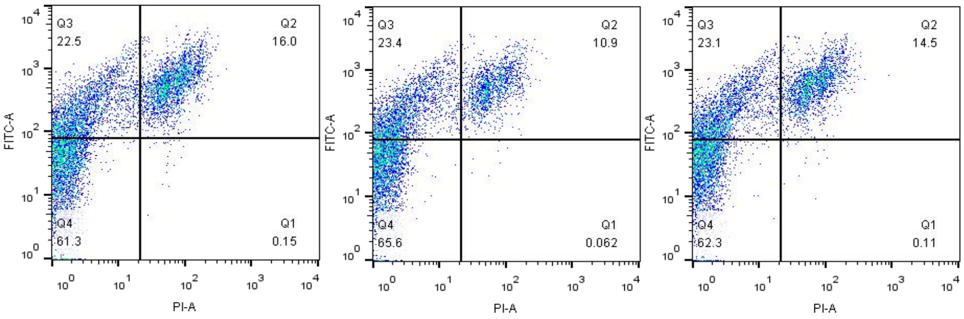

MORE 200 (μg/ml) Group

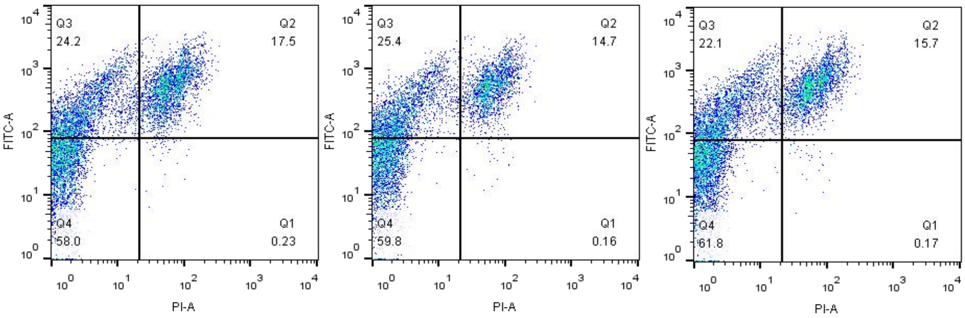

MOHRE 50 (μg/ml)　Group

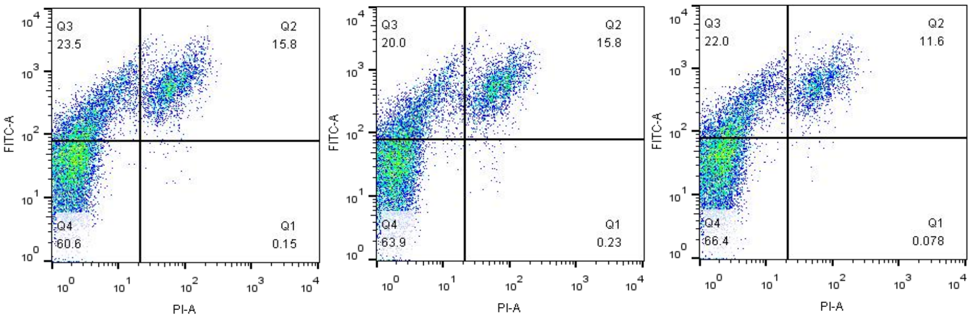

MOHRE 100 (μg/ml)　Group

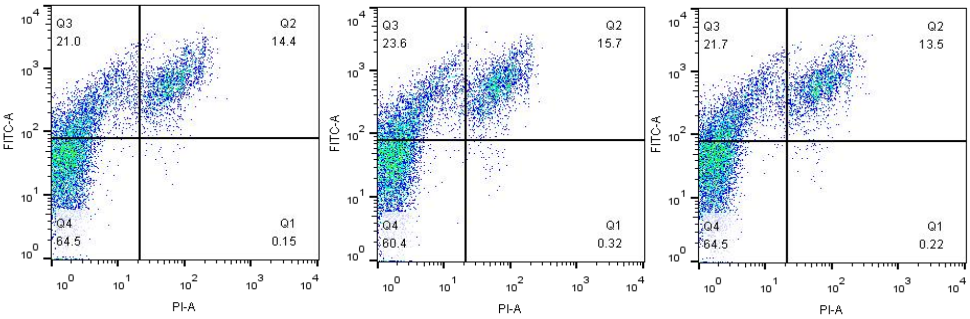

MOHRE 200 (μg/ml)　Group

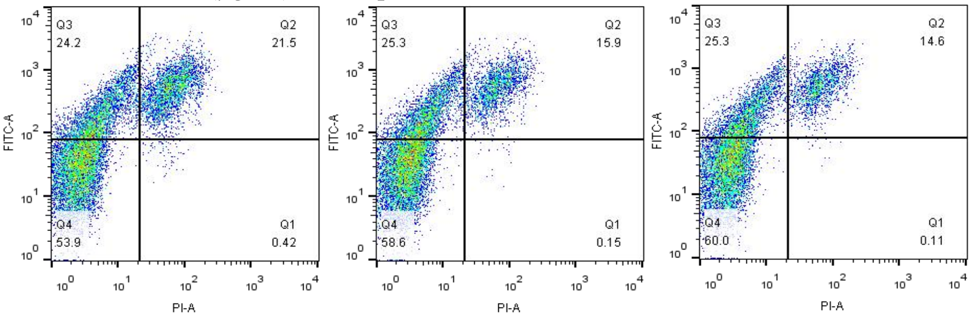

Supplementary Fig. 1. The apoptosis effect of MORE and MOHRE on lymphocyte in the presence of ConA stimulation for 24h. We also found that after stimulation with ConA for 24h, both MORE and MOHRE can dose-dependently induce the apoptosis of ConA-activated T lymphocytes
